# The accuracy of a 4-item hydration self-assessment model to classify urine concentration using different cut-offs

**DOI:** 10.1186/s44410-026-00024-y

**Published:** 2026-04-07

**Authors:** Floris C. Wardenaar, Kinta D. Schott, Ryan G. N. Seltzer, Brooke Butterick, Emily Dow, Parker Kooima, Raul Freire, Kyle D. Reid, Zack Stow, Sai Tejaswari Gopalakrishnan, Richard Remington, David Sklar, Gwyneth Gordon, Brent Ruby, Jefferey L. Burgess, Stavros A. Kavouras

**Affiliations:** 1 College of Health Solutions, Athleat Field Lab, Arizona State University, 425 N. 5th Street, Phoenix, AZ 85004 USA; 2 Tonto National Forest, 2324 E. McDowell Road, Phoenix, AZ 85006 USA; 3 Metals, Environmental and Terrestrial Analytical Laboratory (METAL), 650 E Tyler Mall, Tempe, AZ 85281 USA; 4 Montana Center for Work Physiology and Exercise Metabolism, University of Montana, Missoula, MT 59812 USA; 5 Mel & Enid Zuckerman College of Public Health, University of Arizona, 1295 N. Martin Avenue, Tucson, AZ 85724 USA

**Keywords:** Firefighter, Fluid intake, Urine voids, Urine volume, Urine color, Euhydration, Tactical operator or athletes

## Abstract

**Background:**

Hydration is important for health and performance, but accurate self-assessment methods for active populations are limited. The study objective was to determine a model to classify by self-assessment a low vs. high 24-hour urine concentration, and to determine the accuracy of morning vs. afternoon assessments. The study describes the development and validation of a self-assessment model through exploratory modeling, using stepwise logistic regression. The aim was to determine which hydration markers predict urine specific gravity (using two different USG cut-offs: low ≤ 1.012, and high ≥ 1.020), in two intentionally overlapping 24-hour urine collections leading up to a morning and afternoon hydration self-assessment, and to assess its diagnostic accuracy based on the area under the curve (AUC).

**Results:**

A total of of *n* = 62 wildland firefighters (WLFFs), and *n* = 23 recreational athletes (12% female, median age 25 years, with interquartile range of 24–32) were included. The median USG values of the 24-hour urine collections leading up to the morning and afternoon assessments, were 1.011 and 1.012, respectively. The AUC for the final model, which included self-reported fluid intake; urine frequency, volume, and color, to classify low vs. high USG, for low cut-off (≤ 1.012) was fair in the morning (0.72), and good in the afternoon (0.85), and for high cut-off (≥ 1.020) good for morning (0.81) and afternoon (0.86), respectively.

**Conclusion:**

This 4-item hydration self-assessment model classifies a low vs. high USG acceptable, with slightly higher accuracy in the afternoon. This insight may inform targeted hydration monitoring strategies in occupational settings, for example among WLFFs.

**Supplementary Information:**

The online version contains supplementary material available at 10.1186/s44410-026-00024-y.

## Introduction

Hydration is a critical health determinant, important for the health and wellness of wildland firefighters (WLFFs) [1], whose strenuous job exposes them to elevated risks of injury [2], and exertional heat illness [1, 3], as highlighted in occupational epidemiology. Maintaining proper hydration may reduce the incidence of heat illness [4]. Consuming fluid can mitigate heat strain [5], although it is often difficult to replace more than two-thirds of fluid lost during physical activity [6]. Nevertheless, replacing 50–80% of the fluid lost is still likely to reduce core temperature [7]. In addition, optimizing fluid intake during the 24-hour daily cycle is an important strategy for maintaining an effective work output [8], optimizing cognitive performance [9], and maintaining health [10]. Daily water requirement varies from person to person [11]. A limited number of water turnover studies performed in WLFFs show a mean water turnover of 7.0±1.7 L/d [12], 6.7±1.4 L/d [13], 6.6±2.1 L/d [14], and 9.5±1.7L/d [3]. General recommendations for fluid intake for on-the-job WLFFs, that can be out in the field up to 16 h a day, normally range between 500 and 1000 mL per hour [15]. In the end, hydration status is based not only on what is consumed during a work shift, but also during the remaining part of the 24-hour cycle.

Currently, there is no universally accepted recommendation for fluid intake to represent the adequacy of hydration. However, evidence suggests that higher daily water intake, resulting in a low urine concentration, is inversely associated with chronic health outcomes [16]. Of the multiple ways to determine hydration status, assessing blood concentration is especially useful to detect hypohydration or overhydration [17], as the body regulates blood plasma levels within a tight range, from 285 to 296 mOsm/kg [18]. Still, urine concentration is a more practical marker to evaluate fluid intake, with concentration inversely associated with fluid intake [10]. When fluid intake over a 24-hour period does not match fluid loss, the body will retain available fluid, resulting in a higher urine concentration to minimize substantial loss of body water [19]. An elevated urine concentration has been reported in 40–50% of the participating WLFFs in multiple studies [13, 20–22]. Many studies have applied a USG value of < 1.020 to determine euhydration [23], but at the same time, an optimal fluid intake will normally result in an even lower urine concentration, and as such cut-off values for 24-hour urine of < 500 mmol.kg^− 1^ or ≤ 1.012 USG have been suggested to identify this adequate fluid intake [10, 24]. Whereas the higher cut-off is well accepted to determine underhydration identifying whether individuals have consumed sufficient fluids relative to their daily losses [23], the lower cut-off has described in relation to optimal fluid intake [25, 26].

The collection of 24-hour urine is considered the gold standard for assessing urine concentration [10], but this is a cumbersome process [27], and research has focused on determining the best time to collect a single urine sample to determine hydration [28, 29]. It has been suggested that a spot afternoon urine sample might better reflect a 24-hour urine concentration, than a spot morning urine sample [28, 29], but it is unclear if self-assessment in the morning or afternoon, while including a spot urine sample assessment, results in a better accuracy to distinguish a low from a high 24-hour urine concentration. The present study addresses this issue.

A wide range of markers, that could be self-assessed, have been suggested previously, including urine color (Uc), thirst, fluid intake, body mass change, blood pressure, heart rate [30], urine void frequency [31], void duration [32], and fluid intake [10]. Studies have reported the accuracy of self-assessment, such as measuring Uc, as well as the combination of two methods, such as Uc and the number of urine voids per day [31], as acceptable for standardized self-assessment in active and sedentary populations [31–35]. However, the number of false positive and false negative outcomes has been relatively high (up to 38% for one or the other) as part of these assessments [32, 33, 35]. This raises the question of whether a combination of more than two simple hydration self-assessments can optimize hydration self-assessment. Although the literature clearly states that it is important to monitor hydration status [6, 15, 36, 37], data about the accuracy of hydration self-assessment in WLFFs, and other (operational or tactical) athletes are limited.

Therefore, this research study aimed to determine the best combination of easy to self-assess hydration markers for predicting a low vs. high urine concentration. In addition, the accuracy for performing morning and afternoon assessments compared to 24-hour urine concentration leading up to these assessments, was determined in a group of WLFFs and recreative athletes. Furthermore, this study allowed to explore the use of a mix of self-assessments as a practical tool to support hydration surveillance in active populations.

## Methods

### Research design

From spring to fall of 2024, a multi-level approach was used to validate the best combination of hydration self-assessment markers to predict a low vs. a high urine concentration. Most of the data collection took place at WLFF stations across the Tonto National Forest in spring during the “office period” of the district critical training in which they were not responding to official fire calls. The remainder of the participants consisted of an active surrogate population that was tested at our lab facility in Tempe, Arizona, in the USA. Data (i.e., bodyweight, urine, and self-assessments) were collected during a three-day period, but participants collected their urine on the first two days for a period of 32-hours. All participants were instructed to not alter their fluid intake during the study. Samples were collected in such a way that the concentration could be determined for both the 24-hour leading up to the morning and the afternoon assessment. The 24-hour urine reference periods were intentionally overlapping to align each self-assessment (morning and afternoon) with the physiologically relevant 24-h period preceding that assessment. After collecting data, exploratory modeling determined which variables significantly contributed to predicting a low vs. a high 24-hour urine concentration for morning and afternoon assessments.

### Participants

The study aimed to include as many of the 200 WLFFs working at the Tonto National Forest as possible, and if needed, a sample of surrogate populations physiologically representing the WLFF population during the first part of their annual training phase. During this phase, firefighters performed a substantial amount of office work and averaged 1–3 hours of daily physical activity, including conditioning, brush clearing, and preparing areas for prescribed burns. The surrogate population included active recreational athletes who were reporting training sessions for at least 1–2 h a day for 5 days per week. Inclusion criteria for all participants were being 18–65 years of age regardless of sex, and a self-reported stable weight for the last month (< 10 lbs. fluctuation). Exclusion criteria were the use of thyroid medication, bariatric surgery, cardiovascular disease, renal disease, hepatic disease, a low body weight < 110 lbs. to control for factors that could bias hydration markers, as well as any injury that would not allow physical performance or activity, and being pregnant or lactating. None of the participants reported using diuretics; dietary supplements use was registered, but its use was not an exclusion criterium.

The study was approved by the institutional review board of Arizona State University (STUDY00018531) and the Department of Homeland Security (DHS) Compliance Assurance Program Office (HSR-23-088). The study was performed in accordance with the ethical standards laid down in the 1964 Declaration of Helsinki and its later amendments. Participation was voluntary, and participants read and signed informed consent before the start of the study. Due to federal regulations, no incentives were provided to participating WLFFs, but a $125 incentive was provided to the recreational athletes.

### Protocol

Before data collection, participants were instructed to eat and drink normally, without attempting weight loss or gain throughout the study. No intervention was performed aside of participating in each of the measurements described in the section below.

**Fig. 1:**
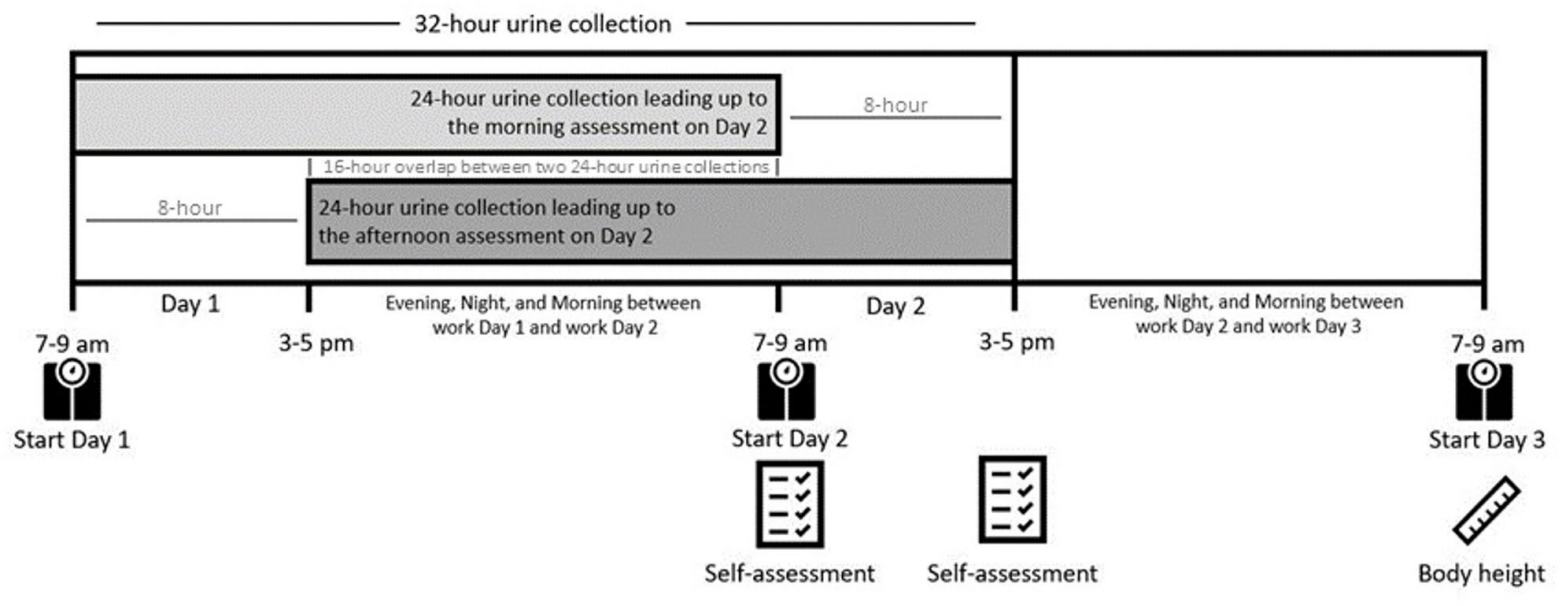
Study timeline for days 1–3 showing a 32-hour urine collection that yielded two intentionally overlapping 24-hour samples. The first was compared with morning hydration self-assessments, and the second with afternoon assessments

Participants collected each separate urine voids in individual containers for a total of 32-hours, starting between 7 and 9 am on the morning of Day 1 and finishing at 3–5 pm on Day 2 (Fig. 1).

This allowed to assess the urine volume and concentration for the first 24 hour leading up to the morning assessment on Day 2, as well as the volume and concentration for the 24-hour leading up to the afternoon assessment on Day 2. The two 24-hour urine samples overlapped intentionally to align each self-assessment (morning vs. afternoon) with a true 24-hour biological reference period. This allowed for comparing the accuracy for morning and the afternoon self-assessment with the real 24-hour urine concentration leading up to each measurement. Sensitivity analyses examined differences and correlations in the underlying 24-hour urine concentrations and related hydration indicators (e.g., urine volume, void duration and total number of voids) between the corresponding morning and afternoon measurement periods.

Participants were instructed to record their fasted morning body weight directly after waking and using the restroom, to determine baseline body weight (and variation) on the mornings of study Days 1, 2, and 3. Participants performed simple hydration self-assessments on Day 2 in the morning (between 7 and 9 am) and afternoon (between 3 and 5 pm).

### Measurements

#### Body weight

During Day 1–3, participants collected their fasting semi-nude morning body weight at home (Digital Body Weight Bathroom Scale, Moss & Stone, New York, NY, USA) after they provided their urine sample.

### 32-hour urine collection reported by participants (Day 1 and Day 2)

#### Urine collection voids

All separate voids throughout Day 1 and Day 2 were collected in 946 mL (32 fl. oz.) containers. At the end of the first test day (Day 1), participants were provided with seven containers for urine collection at home, and were instructed to collect the first morning void and the last afternoon void on Day 2 in two separate black 946 mL (32 fl. oz.) containers to ensure participants were not able to see the urine color of their sample before the urine color assessment. Participants were instructed to write the time of collection on the label of each container.

#### Self-reported number of urine voids

To track urination frequency, participants were given a handheld tally counter and asked to press it each time they voided throughout the study period. At the end of the collection window, when they turned in their urine samples at assigned drop-off points within their workplace, they also reported the total number of voids.

#### Self-reported fluid intake

A ready-to-fill-out fluid diary was provided to help track daily fluid intake throughout the study period, after the first 8 h of the urine collection (Day 1), after the following 16 h (covering the evening, and night of Day 1, until breakfast on Day 2), and the last 8 h of the urine collection (during Day 2). Participants were instructed to record all beverages consumed during each of the three study periods, including water, soda, juice, coffee, tea, and other drinks. The diary included common container sizes (e.g., bottles, mugs, camelbacks), with space to add custom volumes. Participants tallied their intake by number of servings and calculated total fluid volume accordingly. Fluids from food (e.g., soups, fruits) were not included unless consumed primarily as a source of hydration.

### Hydration self-assessment after 24-hours and 32-hours of urine collection (Day 2)

#### Thirst sensation

Participants recorded thirst sensation by: (1) Answering the question: “If you had a glass of water right now, would you feel the need to drink water from it?” (yes/no), and scoring a visual analogue scales (VASs) to assess thirst perception. The visual analog scales consisted of a 175-mm line with an anchor on the left side (0 mm, ‘‘not at all’’) and a second anchor on the 125-mm mark labeled “extremely”. The “extremely” label (125 mm) was described as “the most they had ever felt in their life”. If participants experienced a perception greater than they had ever felt, they could mark beyond the right bound of 125 or extend the line beyond 175 mm; [38] (2) Answering the question: “How intense is your thirst at the moment?”; and (3) “How distressing (bothersome) is your thirst at the moment?” The results were then calculated as a percentage from the 0–175 mm anchors, with “not thirsty at all” up to “extremely thirsty”, and scores ≤ 40% of the VAS scale were considered as not thirsty.

#### Urine void duration of their first morning and afternoon sample

Participants timed the duration of their complete first morning and final afternoon urine spot sample voids in seconds while using their watch or a phone timer. A duration ≥ 16 s indicated a larger urine volume, as determined in a previous study [32].

#### Urine color

A total of 30 mL from the first morning and the last afternoon samples collected in the black containers, leading up to the self-assessment, were transferred to a centrifuge tube with transparent lid by the research team to allow for Uc scoring. For this purpose, two color charts were used: (1) A earlier validated paper 7-color Uc chart of our design [39]. Participants compared their urine sample with each color on the chart while sliding the sample over the white backdrop that was part of the chart (43.2 × 27.9 cm). To control for background lighting, a flashlight with six LEDs (Ozark Trail, Ozark, AR, USA) giving 1848 lx when filtered by a single layer of white masking tape was used. Participants held the flashlight directly underneath the 30 mL centrifuge tube with clear cap while comparing the color of the sample with the chart. (2) A three-dimensional (3D) 7-color model also developed by our research group [39], consisting of 30 mL centrifuge tubes with transparent lids with colors matching those on the aforementioned paper urine color chart. Participants directly compared their urine sample in a 30 mL centrifuge tube with each color on the 3D chart, while being allowed to pick up each of the 3D color references. Participants performed both scoring methods by recording their score on a sheet, and the research team alternated the order of the methods randomizing the order of morning and afternoon assessment for each participant.

### Additional investigator-based data collection, registration, analyses, and calculations

The research team separately recorded markers that potentially could be included in a hydration self-assessment model, such as body weight, number of urine samples, urine volume throughout the collection period, as well as blood pressure and heart rate (after being seated in a chair for 10 min using a digital blood pressure monitor [with blood pressure ± 3 mgHg, and heart rate ± 5 bpm, using the BP7100 blood pressure monitor, OMRON Healthcare, Inc., Kyoto, Japan]), during the self-assessments on Day 2.

#### Body height and BMI

During Day 3, body height was measured by the research team (213 portable stadiometer, SECA, Hamburg, Germany). Body Mass Index (BMI) was calculated by dividing body weight in kilograms by the square of height in meters (kg/m²).

#### Body weight change from baseline

The average body weight of Day 1 and 3 was compared to the Day 2 body weight, in which a 1% decrease from ‘baseline” suggested a large deviation that could indicate underhydration.

#### Urinalysis – urine void frequency, volume, and concentration

Urine void frequency was calculated based on the total number of urine containers each participant handed in during the 32-hour period. Participants wrote the collection time on each cup, allowing the research team to calculate the number of voids for the first 24-hour and the partially concurrent second 24-hour. Urine volume was measured using a kitchen scale within ≤ 0.5-gram accuracy in 0.1-gram increments, assuming that 1 g of urine was equal to 1 mL. Urine specific gravity (USG) was measured onsite for all samples including the spot urine samples that were used as part of the morning and afternoon assessment (4410 PAL10S refractometer, ATAGO, Tokyo, Japan), using previously described standardized procedures at room temperature [40]. The 24-hour USG was calculated as the volume-weighted mean of all samples: $\sum\left(\mathrm{USG}_\mathrm{i}\:^{*}\:\mathrm{Volume}_\mathrm{i}\right)/\:\sum\:\mathrm{Volume}_\mathrm{i}$, in which _i_ indicates the number of samples.

#### Questionnaire

Personal demographics recorded included age, sex, race, ethnicity, work location, work status (incumbent vs. new, year vs. seasonal contract), short medical history, use of medications (with a focus on medication impacting hydration status, such as diuretics), use of dietary supplements, and alcohol and tobacco use.

#### Environmental measurements

During all study days, local weather conditions were measured using a Tempest Weather Station (WeatherFlow-Tempest Inc., Santa Cruz, CA, USA) mounted on a stand at chest height to collect ambient temperature and relative humidity.

### Finalizing the hydration self-assessment model

After data collection, explorative analysis was applied to both continuous (when relevant) and binary outcomes, as listed in the above section. The binary variables, received a score based on a literature based consensus pre-defined cut-off, suggesting a low (0) or high (1) urine concentration. Modeling outcomes were compared with the actual 24-hour urine concentration leading up to the morning and afternoon assessment, categorized as a low urine concentration (USG ≤ 1.012 “optimal fluid intake”) vs. a higher urine concentration (USG ≥ 1.013, “suboptimal fluid intake”).

A stepwise logistic regression was run on each of the variables in the dataset (both binary, and relevant continuous) while controlling for age, sex, race, ethnicity, annual or seasonal hire, BMI, nutritional supplement use, medicine use, alcohol use, nicotine use, dietary supplement use, and environmental conditions, to determine which questions were related to the binary outcome of a low vs. high urine concentration. All variables that were not significantly related to the outcome were dropped from the final model. For this analysis, *p* ≤ 0.10 was the initial threshold for retaining variables in the model, as well as odds ratio 95% confidence intervals not containing the value 1.0, when dealing with categorical outcome variables. A final logistic regression model was run on the full dataset in which variables *p* < 0.05 were retained, with causal priority being granted to variables deemed practically relevant for self-assessment based on the team’s experience in the field of hydration self-assessment. Adjusted model parameter estimates were used to evaluate the unique variance contribution of each variable. These metrics were evaluated to ensure each variable contributed unique variance and predictive value above and beyond all other variables in the model.

The cut-offs of the variables that were selected as part of the final model are: (1) Fluid intake ≥ 2697 mL [91.2 fl. Oz.] in females, ≥ 3697 mL [125 fl. Oz] in males suggesting a low urine concentration vs. <2697 mL [91.2 fl. Oz] in females, or < 3697 mL [125 fl. Oz] in males suggesting a high urine concentration [41]. (2) Urine sample collected before assessment ≥ 250 mL suggesting a low urine concentration vs. <250 mL suggesting a high urine concentration [32]. (3) A 24-hour urine frequency ≥ 7 suggesting a low urine concentration vs. <7 suggesting a high urine concentration [31]. (4) A urine color with a shade ≤ 2 suggesting a low urine concentration vs. ≥3 suggesting a high urine concentration [42].

### Statistical analysis

General characteristics are reported as frequencies (% and n) or median and interquartile range (IQR), as self-reported outcomes were not normally distributed. Descriptive outcomes are reported for the total group or for the individual assessments (i.e., morning vs. afternoon).

As the studies objective was to determine whether the same self-assessment model performs differently depending on the time of day in the same individuals, under near-identical physiological conditions, the overlap between 24-hour urine collection allowed isolation of the time-of day effect and simple repeated measure testing was performed. Differences between morning and afternoon assessments for continuous or scale-based variables were analysed using Wilcoxon signed-rank test or Mann Whitney U tests for continuous variables (with a Rank Biserial Correlation effect size: *r*_*rb*_, calculated using the difference between the sums of positive (W⁺) and negative (W⁻) ranks, according to the formula r_r_b = (W⁺−W⁻)/(W⁺+W⁻), with small (≥ 0.1), medium (≥ 0.3), and large (≥ 0.5) effect sizes. Differences for categorical variables were analyzed using Pearson Chi-Square tests or Fisher’s Exact tests (with Cramer’s V effect size: V, calculated as calculated as √(χ² / [n × (k − 1)]), in which χ² is the Chi-square test statistic, n the total sample size, and k the smallest dimension of the cross-table), with small (< 0.1), medium (0.1–0.3), and large (> 0.3) effect sizes. Spearman correlations (*r*_*s*_), reported as positive correlations, were calculated to identify the strength of the relationship between variables, with Cohen’s-d effect size: small (≤ 0.20), medium (0.21–0.79), and large (≥ 0.80). Significance was set at *p* ≤ 0.05.

Finally, the fit for the model and its individual variables for morning and afternoon for the ≤ 1.012 USG cut-off, as well as preliminary sensitivity analysis for a ≥ 1.020 USG cut-off, was assessed by calculating the area under the curve (AUC), as well as true positive (TP), false positive (FP), false negative (FN), and true negative (TN) scores. These values were used to calculate accuracy (TP + TN/Total), sensitivity (TP/TP + FN), and specificity (TN/TN + FP). When interpreting the results, AUC was considered excellent (≥ 0.90), good (0.80–0.89), or fair (0.70–0.79). Sensitivity and specificity scores are preferred to be above 0.80. Sensitivity is defined as the number of TP scores suggesting underhydration, divided by the sum of TP and FN scores. Specificity is defined as the number of TNs divided by the sum of FPs and TN.

Based on a known estimated underhydration incidence in a general athletic population of 35% [42], and an expected maximal underhydration level in WLFs of 50%, with an alpha of 0.05 and power of 80%, a sample size of *n* = 82 was targeted.

## Results

As shown in Table 1, a total of 85 participants were included (of which 12%, *n* = 10, were female). The group consisted of *n* = 62 WLFFs and *n* = 23 recreational athletes. Data for WLFFs was collected during their district critical training, and therefore no actual firefighting activity was performed during the study period. The median age was 26 [24–32] years, with body mass of 81.0 kg [71.3–88.3] and BMI 25.7 [23.9–28.9]. The total group represented various races, but no Black/African American participants were enrolled in the study. A total of 19% (*n* = 16) identified as Hispanic or Latino. Moderate alcohol consumption was reported (65%, *n* = 55), with on average 1 [0.0–3.0] consumption a week, and 77% of the participants used some form of nutritional supplements. Supplement use included vitamins and minerals (48%, *n* = 41), sports drinks (42%, *n* = 36), and energy drinks (32%, *n* = 27). Only 13% (*n* = 11) of the paricipants reported some form of medication. During data collection days, median air temperature for the various sites was 18.6 °C [17.3–27.5], with relative humidity of 29.0% [29.0–50.0].

**Table 1:** Descriptive demographics for included participants (*n* = 85)

	Demograpics
Metrics	
Age (years)	26 [24–32]
Body height (cm)	176 [170–182]
Body mass (kg)	81.0 [71.3–88.3]
Body mass index (BMI)	25.7 [23.9–28.9]
Type of participants	
Wildland Firefighter (%, n)	73% (62)
Recreational athlete (%, n)	27% (23)
Sex % (n)	
Male	88% (75)
Female	12% (10)
Race % (n)	
American Indian/Alaska Native	4% (3)
Asian	23% (19)
Black/African American	--
Native Hawaiian/Pacific Islander	1% (1)
White	65% (54)
American Indian/Alaska Native & White	4% (3)
Asian & White	4% (3)
Ethnicity % (n)	
Hispanic/Latino	19% (16)
Contract % (n)	
1 + 3 Year	36.5% (31)
2 + 4 Seasonal	36.5% (31)
5 NA	27% (23)
Status % (n)	
1 Hotshots	29% (25)
2 Engine crew	44% (37)
5 NA	27% (23)
Habits	
* Alcohol*	
Consuming alcohol (%, n)	65% (55)
Servings a week (#)	1.0 [0.0–3.0]
* Nicotine*	
Using some form of nicotine (%)	39% (32)
Cigarette/Cigar or equivalent (%)	2% (2)
Vape (%)	4% (3)
Chew (%)	5% (4)
Gum (%)	4% (3)
Patch (%)	--
Other: Pouches (%)	29% (25)
* Dietary supplements*	
Use at least one supplement (%)	77% (65)
Vitamins & minerals (%)	48% (41)
Protein (%)	58% (49)
Energy drink (%)	32% (27)
Sports drink (%)	42% (36)
Pre-workout (%)	12% (10)
Other (%)	8% (7)
Medication % (n)	
Using at least one type of medication	13% (11)
Prescription	7% (6)
Over the counter (no prescription)	5% (5)
Contraception	2% (2)

### Descriptive urine-based outcomes

The 24-hour USG for the first 24-hour (1.011 [1.008–1.016]) and the second 24-hour (1.012 [1.008–1.016]) of the intentionally overlapping reference periods were not significantly different (*p* = 0.465). On the other hand, spot morning urine USG was higher (1.018 [1.013–1.023]) than in the afternoon (1.011 [1.006–1.021]), with *p* < 0.001, and a moderate r_rb_=-0.29, as shown in Table 2.

USG was highly correlated between the first and second 24-hour urine collection (*r*_*s*_=0.887, *p* < 0.001). At the same time, the correlations between spot morning USG and the first 24-hour USG (*r*_*s*_=0.613, *p* < 0.001) was moderate. In addition, spot afternoon USG showed a higher correlation for the partially concurrent second 24-hour USG (*r*_*s*_=0.670, *p* < 0.001).

Contingency table analysis positioning the 24-hour urine concentration vs. the spot urine sample USG categorized for a low vs. high urine concentration revealed spot morning samples in the morning and in the afternoon significantly differed from the 24-hour urine samples. The measured USG of the spot morning and afternoon urine samples, similarly classified the urine sample concentration as low vs. high against the “gold standard” 24-hour urine collections with an accuracy of 58.0% (low cut-off, ≤ 1.012 USG), and 70.4% (high cut-off, ≥ 1.020) in the morning vs. an accuracy of 77.6% (low cut-off. ≤1.012 USG), and 79.2% (high cut-off, ≥ 1.020) in the afternoon.

Further shown in Table 2, is that the average urine volume (~ 2175 mL) was not significantly different between the two 24-hour collections (*p* = 0.685). Void duration of spot urine samples was roughly 5 seconds longer in the morning (20.5 [13.1–30.0] sec.) vs. afternoon (15.0 [9.5–20.0] sec.), with *p* < 0.001. At the same time, there was no difference between the number of voids (7 [5–9], *p* = 0.464) in each of the 24-hour leading up to the morning and afternoon assessments. Finally, there was a high (*r*_*s*_:0.85, *p* < 0.001) and moderate (*r*_*s*_:0.73, *p* < 0.001) correlation between the individual number of samples collected by the research team (Table 2), and self-reported urine voids for morning and afternoon assessment (Table 3), respectively.

**Table 2:** Measured descriptive outcomes for urinalysis (median and IQR) for the morning and afternoon, and the difference between time points (*n* = 85)

	Morning	Afternoon	*P*-value, Effect size
Urine concentration			
24h sample USG leading up to the self-assessment	1.011 (1.008–1.016)	1.012 (1.008–1.016)	*p* = 0.465, r_rb_= 0.12
Spot morning sample USG	1.018 (1.013–1.023)	1.011 (1.006–1.021)	*p* < 0.001, r_rb_= -0.29
Urine volume			
24h sample leading up to the self-assessment (mL)	2131 (1444–3283)	2248 (1620–3186)	*p* = 0.685, r_rb_= 0.11
Spot sample (mL)	450 (291–611)	250 (128–406)	*p* < 0.001, r_rb_= -0.53
Void duration			
Spot sample (sec)	20.5 (13.1–30.0)	15.0 (9.5–20.0)	*p* < 0.001, r_rb_= -0.38
Number of voids			
24h leading up to the self-assessment(#)	7 (5–9)	7 (5–9)	*p* = 0.464, r_rb_= 0.19

*P*-value was set at *p* ≤ 0.05. The effect size was calculated as a rank-biserial correlation (r_r_b) using the difference between the sums of positive (W⁺) and negative (W⁻) ranks, according to the formula r_r_b = (W⁺−W⁻)/(W⁺+W⁻), with small (≥ 0.1), medium (≥ 0.3), and large (≥ 0.5) effect sizes. A negative effect size indicates that the morning value is larger than the afternoon value

### Descriptive self-reported outcomes

As noted in Table 3, Uc self-assessment was significantly different between the morning and afternoon for both of the methods used (Printed color chart: 2 [2–3] vs. 2 [1–3], and 3D color chart: 2 [2–4] vs. 2 [1–3]), with *p* ≤ 0.006. Urine void frequency for the first (7 [5–9]) and second (7 [5–8]) 24-hour period was signifcantly different, as a result of a lower frequency from the 75th percentile and up, during the second period (*p* = 0.021). All of the markers for thirst were scored higher in the morning than in the afternoon (*p* ≤ 0.001). For example, more participants reported that they would drink water in the morning (76%) vs. afternoon (55%). At the same time total fluid intake (~ 4300 mL) was not significantly different for both 24-hour periods (*p* = 0.060). Body mass change (%) from a 2d baseline (only calculated based on the morning bodyweight assessment) was small (0.01 [-0.43-0.46] %), and only 14.9% of the participants reported ≥ 1% change, but no changes above 2% body mass were reported. Borderline significant differences were reported for blood pressure, with small effect sizes, and a significant difference with a moderate effect size was seen for heart rate between morning and afternoon assessments as shown in Table 3.

**Table 3:** Self-assessed and measured hydration markers categorized by morning and afternoon, and the difference between time points (*n* = 85)

	Morning	Afternoon	*P*-value, Effect size
*Self-assessment*			
* Urine color*			
Printed color chart (#)	2 (2–3)	2 (1–3)	*p* = 0.006, r_rb_= -0.36
3D color chart (#)	2 (2–4)	2 (1–3)	*P <* 0.001, r_rb_= -0.66
* Urine void frequency*			
Last 24-hour (#)	7 (5–9)	7 (5–8)	*p* = 0.021, r_rb_= -0.21
* Thirst*			
Drink water (%, n) (*n* = 83)	76 (62)	55 (41)	*P* < 0.001, V = 0.42
Thirst intensity (VAS, calculated to %)	34.3 (21.4–64.0)	21.4 (10.0-33.6)	*P* < 0.001, r_rb_= -0.38
Thirst distress (VAS, calculated to %)	21.4 (11.8–30.7)	12.1 (3.6–23.6)	*p* = 0.001, r_rb_= -0.33
* Fluid intake*			
Last 24-hour (mL)	4377 (2839–5463)	4259 (2906–5486)	*p* = 0.060, r_rb_= -0.23
Other measurements			
* Body mass change from 2d baseline*			
Percent change (kg)	0.01 (-0.43-0.46)	--	--
More than 1% change (%)	14.9 (11)	--	--
* Vitals*			
Systolic blood pressure (mmHg)	124 (118–140)	126 (116–135)	*p* = 0.059, r_rb_= -0.20
Diastolic blood pressure (mmHg)	76 (69–83)	72 (67–79)	*p* = 0.045, r_rb_= -0.19
Heart rate morning (bpm)	69 (62–78)	72 (68–83)	*P* < 0.001, r_rb_= 0.31

### Explorative modeling to determine predictors to self-assess a low urine concentration

For the first step of the explorative modeling to determine which outcomes were able to predict a low vs. high urine concentration while using the low cut-off (≤ 1.012 USG), all self-reported and measured variables were used. After this a total of 12 models were run (6 for the morning and afternoon, respectively), added as Supplementary File 1, revealing good areas under the curve (AUC) for the combined continuous or binary variables categorized as self-reported or measured. The USG based AUC ranged from 0.794 to 0.968 in the morning vs. 0.739–0.929 in the afternoon. Individual variables that consistently and significantly contributed two times or more to these 12 models were: Self-reported fluid intake (#4), self-reported urine void frequency (#2), and urine volume first-morning or afternoon sample (#4), thirst distress (#2). Blood pressure (systolic and diastolic) in the morning (#1), contributed only once. Multiple covariates, such as age, sex, race, ethnicity, annual or seasonal hire, BMI, nutritional supplement use, medicine use, and environmental conditions were included with the final model, but none of them were significantly related to the outcome (*p* > 0.05) or confounded (changed the significance level of) the existing significant relationships between the other predictor variables and the outcome.

### Accuracy of single and combined variables to self-assess a low urine concentration

The final 4-variable model presented in this article resulted in the practical best fit to distinguish a low from a higher urine concentration with an AUC of 0.72 and 0.81 for morning, and 0.85 and 0.86 for afternoon assessments, for the low (≤ 1.012 USG) and high (≥ 1.020 USG) cut-offs respectively. This final model was based on self-reported fluid intake, urine void frequency, morning void urine volume, and urine color with cut-offs applied as described in the method section of this article. Each outcome was assigned with a score 0/1, with a score of 0–1 suggesting a low urine concentration (i.e., optimal fluid intake) vs. a combined score 2–4 suggesting a high urine concentration (i.e., suboptimal fluid intake). The results for the outcome of this model, as well as each of the individual contributing variables, are reported in Table 4.

The AUC for each of the individual variables, regardless of low or high cut-off, ranged from 0.53 to 0.71, but were inferior when compared to the total group model regardless assessment time or selected cut-off. The raw analysis output for each of these models can be found in Supplementary File 2.

**Table 4:** Morning and afternoon assessment receiver operating characteristic evaluation of the four-variable model, individual variables, and the accurate percentage of correct classification of low vs. high 24-hour USG (≤ 1.012 or ≥ 1.020)

Assessment	Variables	AUC	Sensitivity %	Specificity %	TP % and *n*	TN % and *n*	FP % and *n*	FN % and *n*
Morning USG 1st 24 h ≤ 1.012	TOTAL GROUP: 4-variables: intake/frequency/volume/Uc	0.72	66.7	62.5	40.0 (32)	25.0 (20)	15.0 (12)	20.0 (16)
	1.Self-reported fluid intake (intake)	0.64	66.7	53.1	40.0 (32)	21.3 (17)	18.8 (15)	20.0 (16)
	2.Self-reported urine void frequency (frequency)	0.65	68.8	59.4	41.3 (33)	23.8 (19)	16.3 (13)	18.8 (15)
	3.Urine volume first-morning sample (volume)	0.53	85.4	21.9	51.3 (41)	8.8 (7)	31.3 (25)	8.8 (7)
	4.Self-reported 3D Uc color chart score (Uc)	0.58	60.4	53.1	36.3 (29)	21.3 (17)	18.8 (15)	23.8 (19)
Afternoon USG 1st 24 h ≤ 1.012	TOTAL GROUP: 4-variables: intake/frequency/volume/Uc	0.85	73.7	75.9	41.8 (28)	32.8 (22)	10.4 (7)	14.9 (10)
	1.Self-reported fluid intake (intake)	0.71	84.2	58.6	47.8 (32)	25.4 (17)	17.9 (12)	9.0 (6)
	2.Self-reported urine void frequency (frequency)	0.63	63.2	62.1	35.8 (24)	26.9 (18)	16.4 (11)	20.9 (14)
	3.Urine volume afternoon sample (volume)	0.67	68.4	65.5	38.8 (26)	28.4 (19)	14.9 (10)	17.9 (12)
	4.Self-reported 3D Uc color chart score (Uc)	0.68	86.8	51.7	49.3 (33)	22.4 (15)	20.9 (14)	7.5 (5)
Morning USG 1st 24 h ≥ 1.020	TOTAL GROUP: 4-variables: intake/frequency/volume/Uc	0.81	59.7	87.5	64.2 (43)	10.4 (7)	1.5 (1)	43.3 (29)
	1.Self-reported fluid intake (intake)	0.66	59.7	50.0	64.2 (43)	6.0 (4)	6.0 (4)	43.3 (29)
	2.Self-reported urine void frequency (frequency)	0.69	61.1	75.0	65.7 (44)	9.0 (6)	3.0 (2)	41.8 (28)
	3.Urine volume first-morning sample (volume)	0.59	84.7	37.5	91.0 (61)	4.5 (3)	7.5 (5)	16.4 (11)
	4.Self-reported 3D Uc color chart score (Uc)	0.68	58.3	75.0	62.7 (42)	9.0 (6)	3.0 (2)	44.8 (30)
Afternoon USG 1st 24 h ≥ 1.020	TOTAL GROUP: 4-variables: intake/frequency/volume/Uc	0.86	60.3	90.0	52.2 (35)	13.4 (9)	1.5 (1)	34.3 (23)
	1.Self-reported fluid intake (intake)	0.65	70.7	60.0	61.2 (41)	9.0 (6)	6.0 (4)	25.4 (17)
	2.Self-reported urine void frequency (frequency)	0.68	58.6	80.0	50.7 (34)	11.9 (8)	3.0 (2)	35.8 (24)
	3.Urine volume afternoon sample (volume)	0.67	60.3	80.0	52.2 (35)	11.9 (8)	3.0 (2)	34.3 (23)
	4.Self-reported 3D Uc color chart score (Uc)	0.72	77.6	70.0	67.2 (45)	10.4 (7)	4.5 (3)	19.4 (13)

The combined (*n*) outcome for TP/TN/FP/FN equals the total n for each line

## Discussion

A 4-item hydration self-assessment model including self-reported fluid intake, urine frequency, morning urine volume, and morning urine color resulted in the best combination to determine a low vs. a higher USG. In addition, the accuracy of hydration self-assessment to determine a low vs. higher USG was slightly higher in the afternoon. Finally, applying a low (≤ 1.012) vs. higher (≥ 1.020) USG cut-off showed similar accuracy specifically for the afternoon with an AUC ranging from 0.85 to 0.86, whereas the morning showed a larger range from 0.72 to 0.81 for the low and higher cut-off, respectively.

### Morning vs. afternoon hydration self-assessment accuracy

The USG values of the urine collected in this study were equally distributed around the low cut-off (≤ 1.012), providing the best possible distribution to assess the accuracy of the 4-item hydration self-assessment model to determine a very low urine concentration, suggesting an optimal fluid intake [25, 26]. At the same time, the higher USG cut-off (≥ 1.020) showed a very similar accuracy, but as the data was fairly skewed towards lower values generalization of this result needs to be done with caution. Although the 24-hour collections are not statistically independent, independence between measurements was not required to address our research question. The purpose was to evaluate within-subject differences in diagnostic accuracy between time points, rather than to test independent samples. Any within-subject correlation would, if anything, slightly reduce variability and increase precision of the comparison. Regardless of the selected cut-off (i.e. low vs. high), the accuracy was the highest in the afternoon. Earlier research revealed that a spot-afternoon sample fairly well reflects a 24-hour urine concentration [28, 29], and now this study also shows that hydration self-assessment, when applying the same method, is more accurate in the afternoon than in the morning.

### Performance of the 4-item model relative to single variable measures

Each of the four variables selected for this model can be considered discriminatory to determine hydration supported by substantial scientific evidence. For example, the model scores fluid intake to be lower than vs. meeting or higher than the recommended intake [41]. Additionally, the cut-off volume for the urine sample (≥ 250 mL) included in this model was based on an earlier reported median volume measured in an athletic population providing spot urine samples under similar environmental conditions [32]. Furthermore, a 24-hour urine frequency ≥ 7 has been previously indicated to reflect good hydration [31], as well as a urine color with a shade 1–2 that also previously was reported to reflect a low urine concentration [42]. In general, the 4-item accuracy led to a substantially higher correct classification of true positive and true negative samples than any of the individual measurements.

The suggestion to combine measurements to determine hydration is not new. Earlier, a method including the assessment of bodyweight change (W), urine color (U), and thirst (T), abbreviated as “WUT” was proposed as a good way to determine dehydration [43]. Although dehydration often is defined as the active process of losing body water through sweat during physical activity [36], WUT was suggested to be a memory device designed to simplify athlete self-monitoring of day-to-dayhydration status. As such multiple efforts have been put in to place to validate the accuracy of the WUT against an elevated high urine concentration (≥ 1.020 USD), reporting a good specificity to detect a high urine concentration based on a WUT-score 3 (≥ 96%) [44, 45], but a much lower specificity for a WUT-score 2 (57%) [44, 45]. The 4-item model developed as part of this study showed a robust high sensitivity in WLFFs (0.80%) to determine a low urine concentration. The latter may also be important, allowing to confirm an optimal fluid intake with a subsequent low urine concentration, a gap the current study validating the accuracy of the 4-item hydration self-assessment fills. Additionally, it has been shown that the combination of urine frequency during the last 24-hours and a light urine color also has a high accuracy (97%) and sensitivity (100%) to determine a high urine concentration ≥ 800 mmol.kg^− 1^[31]. Somewhat lower numbers were reported for sensitivity (ranging from ~ 60–74%) and specificicty (ranging from ~ 63–90%) in the current study using the 4-item hydration self-assessment model, but the overall accuracy (based on AUC), specifically in the afternoon, ranged 0.85–0.86 for both USG cut-offs, respectively.

### Self-assessment versus investigator-based scoring accuracy

An important difference between this study and most others assessing the impact of assessments such as urine color, is that the outcome for the current study has been based on self-scoring by participants (as relevant in an applied setting), and not on investigator-based assessment [31, 34], which has been shown to provide more accurate outcomes [42]. Further the self-assessment was applied to estimate 24-hour urine concentration, whereas most of the other studies focused on determining the accuracy of assessing urine concentration of spot urine samples [32–34, 44, 45]. During this study, participants used a fluid recording sheet with different fluid serving sizes, and tallied the number servings of fluid consumed, without considering the fluid level of solid foods. Although there was no way to determine the precision of the actual fluid intake estimate, in previous research the total fluid intake has been reported to have a fair positive correlation (*r* = 0.65) with 24-hour urine osmolality [10]. Further, in this case, the investigators measured the volume of the first-morning urine sample at their lab facility, but in real-life this could be easily completed by participants if samples were collected in a measurement cup to determine the volume instantly. In addition, after determining the urine sample volume, transferring a subsample of the urine in a separate 30 mL tube would allow for a more accurate standardized urine color assessment [35]. As urine frequency has been suggested as an important marker to determine urine concentration [31], this study asked participants to use a tally counter to determine the number of total urine voids during a 24-hour period which warranted a high correlation with the actual number of urine containers collected. Further, based on previous work, the volume of ≥ 250 mL suggested as cut-off, related well with a light urine color [42], and this was more accurate than voiding duration [32]. Finally, Uc assessment when scored in a container is more accurate than when scored from the toilet bowl [32, 35]. Additionally, container material and light conditions may influence urine color scoring [35]. Therefore, it has been suggested that urine samples should be transferred to a standardized container before assessing Uc, while adjusting for light conditions to optimize scoring accuracy and test-retest reliability [35]. Finally, although the hydration self-assessment model described in this article includes the 3D Uc assessment, secondary analysis suggests the 3D method performs similarly to the traditional printed Uc chart, likely allowing the two methods to be used interchangeably [39].

### Practical applications

It is important to consider that the newly developed 4-item model, similar to the previously discussed combined variable methods (i.e., WUT and combination of urine frequency and Uc), all require a substantial effort from the person performing the self-assessment. Consequently, this results in improved accuracy than the assessing a single variable. There might be skepticism regarding the real-world feasibility and compliance with tracking multiple variables.

**Fig. 2:**
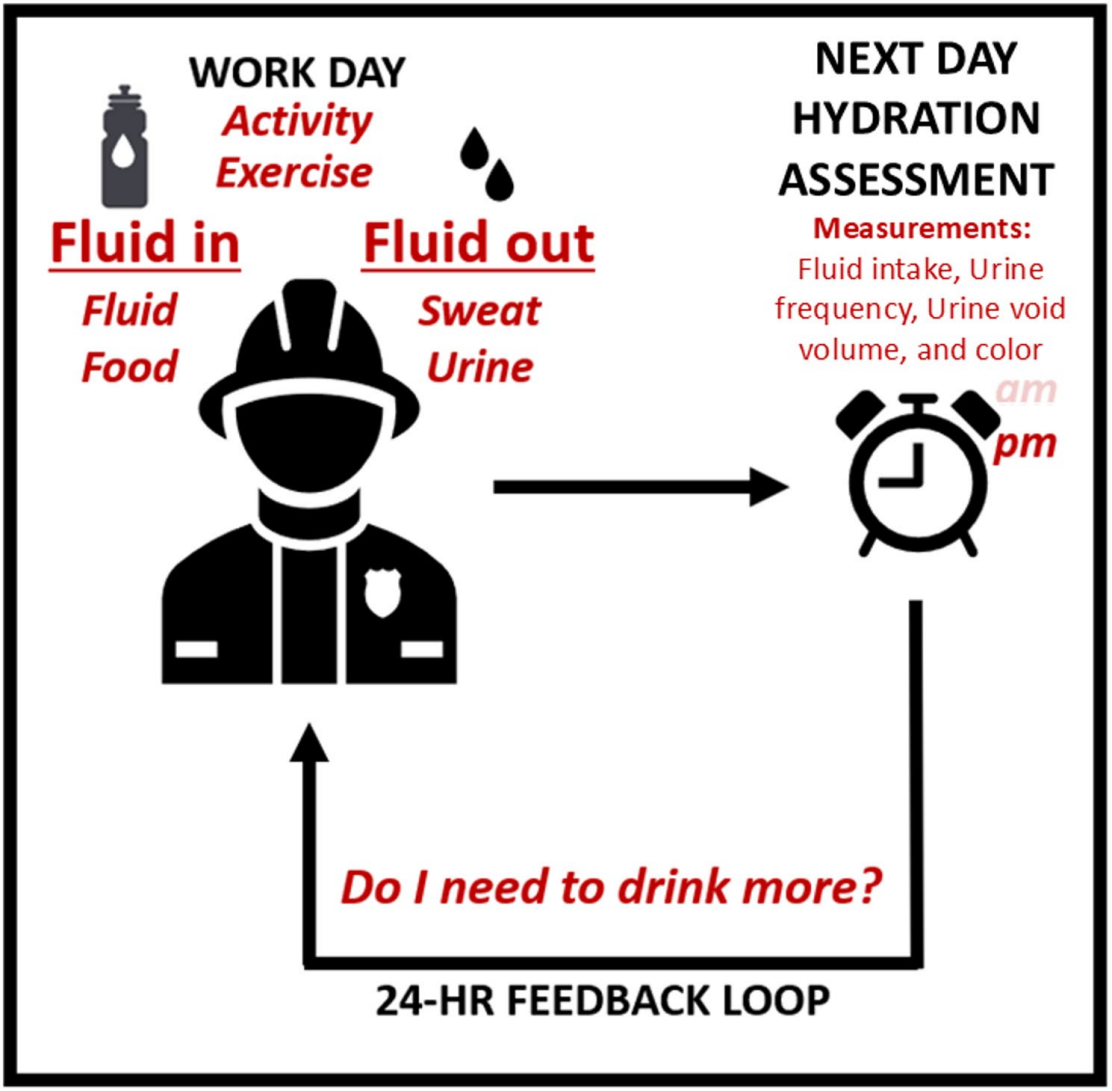
Model for hydration self-assessment in which urine concentration is considered as leading determinant of fluid intake estimated by a combination of four measurements. Assessments may be conducted in the morning or afternoon; however, afternoon sessions are recommended for optimal accuracy

At the same time, hydration assessment in applied settings (i.e., outside the clinic) needs improvement [46]. It has been earlier suggested that there is a need for the development of process-based (i.e., longitudinal, self-monitored) hydration models [16]. The findings from this study suggest that the self-assessment model—regardless of the time during the day—could serve as a feasible and scalable approach to monitor hydration status in occupational cohorts. Traditional methods of hydration assessment, such as full 24-hour urine collection or laboratory biomarker analysis, are often impractical in field-based settings [46]. In contrast, our 4-item model, based on readily observable indicators, offers a practical alternative that may be particularly valuable in occupations with high fluid turnover or limited access to health services, such as wildland firefighting, construction, and agriculture [6, 47]. Importantly, the highest diagnostic accuracy found during this study (AUC = 0.85–0.86 for low vs. high USG cut-off, respectively) supports its relevance for use in epidemiological surveillance of hydration-related health risks and performance outcomes. The 4-item model could be particularly relevant for individuals identified as low fluid consumers, as the integration of these self-assessments into a structured worksheet could provide a visual and individualized representation of hydration markers in relation to daily fluid consumption patterns. Therefore, the next steps to further implement this knowledge include converting this model into a worksheet or other practical format, allowing individuals to regularly assess their hydration status in a simple and structured manner. The method could be used as part of a hydration self-assessment strategy allowing for a 24-hour feedback loop, as shown in Fig. 2. This enables individuals to identify which behaviors—such as low fluid intake or infrequent urination—may require adjustment. Self-monitoring, by linking personal observations with actionable feedback, supports a more individualized approach [48], which could help to achieve optimal hydration. In addition, to translate this model to a field setting, there is a need to properly instruct individuals how to perform the self-assessments, while making sure that the assessment tools are available. In addition, the models efficacy, or in other words the impact of hydration self-assessment on fluid intake, should be evaluated through well-controlled research studies using this novel tool ensuring proper effect evaluation [6, 49]. Finally, the integration of this model as part of routine health and performance monitoring, for example as part of workplace hydration monitoring [50], as part of efforts to optimize hydration related to work performance, thermal stress, injury prevention, or chronic disease risk, should be investigated.

### Strengths and limitations

This study includes clear strengths, such as performing assessments in the morning and afternoon in a real-life setting, with 24-hour urine collections directly leading up to each of these measurements. The intentional overlap between the first and second 24-hour urine collections allowed to reduce variability and avoid the confounding influence of day-to-day fluctuations that could occur with fully independent 24-hour samples. The strong correlation in 24-hour USG values confirms consistency in physiological hydration status, which was necessary to meaningfully compare the accuracy of assessments conducted at different times of day. Additionaly, the study evaluated a wide range of self-scored variables in addition to investigator-based outcomes. Although fluid intake was not measured, it was estimated by participants, but 24-hour USG as a marker of concentration was calculated, and urine concentration has been reported to correlate fairly well (*r* = 0.65) with fluid intake [10]. Another strength of this study was to standardize the Uc assessment as described in the method section, as the accuracy of urine color scoring is impacted by light conditions [35].

At the same time, the study holds various limitations. As the measured population turned out to be very well-hydrated, the investigators are fairly confident that the 4-item hydration self-assessment method performs well for the selected low USG cut-off (≤ 1.012), and although the higher USG cut-off (≥ 1.020) reports similar values its results needs to be interpreted with caution as the data was skewed to the lower end of the cut-off. In this study, body weight change was calculated using the average of Day 1 and Day 3 morning weights as baseline, rather than a fully hydrated multi-day baseline as has been recommended before [51], which may have underestimated the accuracy of this marker in predicting hydration status, and as a result body weight change may not have made it as a meaningful predictor of hydration in the current study. A similar issue may have occurred for thirst, as scores (mm) were converted to percentages based on the full 175-mm VAS (used to reduce ceiling effects). This may have effectively increased the 40% threshold relative to the 125-mm anchor for extreme thirst, potentially reducing the likelihood of classifying participants as thirsty. However, thirst was examined using multiple representations (binary and continuous), and also the continuous measures were not meaningfully associated with 24-hour USG.

Also, data were collected during a WLFF training phase at the start of the season, as such, participants were not actively fighting fires, and maybe the focus on hydration, as well as participating in hydration research, artificially influenced the hydration behavior of the participants. A limited number of females were included, therefore in actuality the percentage of female participants overrepresented the actual female count in the fire service, but underpresented the number of females in the general athletic population. Therefore, no clear differentiation could be made between the accuracy and usability of the method for sex. Additionally, participants may have adjusted their fluid intake as they knew they were assessed.

Finally, the results from the afternoon assessment were based on a slightly smaller sample (as shown in Table 4), which may have affected the reported accuracy.

## Conclusions

The newly developed 4-item hydration self-assessment model, including self-reported fluid intake, urine frequency, morning urine volume, and morning urine color, demonstrated substantially higher accuracy for classifying low versus high 24-hour urine specific gravity (USG) compared with any of the individual model components. Model performance was superior in the afternoon compared with the morning. Although two USG cut-offs (≤ 1.020 and ≥ 1.020) were applied, producing comparable outcomes, results for the higher cut-off should be interpreted with caution due to the limited number of datapoints exceeding this threshold. These insights may inform targeted hydration monitoring strategies in athletic and occupational settings, particularly among firefighters, by providing a practical approach for identifying individuals with suboptimal fluid intake who are motivated to improve their hydration status. Tracking multiple self-reported hydration indicators may further enhance individual awareness of hydration patterns and facilitate behavior change aimed at optimizing daily fluid consumption.

## Supplementary Information

Supplementary Material 1.

Supplementary Material 2.

## Data Availability

The datasets used and/or analyzed during the current study are available from the corresponding author upon reasonable request.

## Abbreviations

3D: Three-dimensional
AUC: Area under the curve
BMI: Body mass index
DHS: Department of Homeland Security
FN: False negative
FP: False positive
HSR: Human Subjects Research (identifier code in DHS approval)
IQR: Interquartile range
NY: New York
SECA: Scale company brand (Hamburg, Germany)
TP: True positive
TN: True negative
Uc: Urine color
USA: United States of America
USG: Urine specific gravity
VAS: Visual analogue scale
WLFF(s): Wildland firefighter(s)
