# Supplementary material for: The accuracy of a 4-item hydration self-assessment model to classify urine concentration using different cut-offs
Source: Perform Nutr. 2026 Apr 7;2(1):8. doi: 10.1186/s44410-026-00024-y (PMC13056763; doi:10.1186/s44410-026-00024-y)
Supplement: Supplementary file 1 — Supplementary Material 1. [file 44410_2026_24_MOESM1_ESM.pdf]

SUPPLEMENTARY FILES

Supplementary File 1

Explorative modeling to determine which outcomes were able to predict a low vs. high urine concentration including all self-reported and measured variables were used (12 models).

This file represents 6 models for the morning assessment, and 6 models for the afternoon assessment for USG.

Morning modeling was performed against the 1st 24h USG, and the afternoon modeling was performed against the 2nd 24-hour USG (≤1.012) categorized as low vs. higher.

Table of contents

MORNING MODEL likely to be self-reported variables (continuous) by participants

MORNING MODEL likely to be self-reported variables (continuous) by participants

MORNING MODEL measured continuous variables by the research team

MORNING MODEL likely to be self-reported binary

MORNING MODEL likely to be self-reported binary

MORNING MODEL measured binary

AFTERNOON MODEL likely to be self-reported variables (continuous) by participants

AFTERNOON MODEL likely to be self-reported variables (continuous) by participants

AFTERNOON MODEL measured continuous variables by the research team

AFTERNOON MODEL likely to be self-reported binary

AFTERNOON MODEL likely to be self-reported binary

AFTERNOON MODEL measured binary

<u>MORNING MODEL likely to be self-reported variables (continuous) by participants</u> (MORNING SR)

OPTION 1 (differentiating between color charts, print color chart) – continuous variables
BW change% based on two days; Drink water morning (yes/no); Thirst intensity morning (percentage); Thirst distress morning (percentage); Self-reported fluid intake first 24-hour (mL); Color chart Paper morning (score 1-7); Urine volume morning sample (seconds); Self-reported urine frequency over 24-hour.

The LOGISTIC Procedure

Model Information		
Data Set	WORK.FEMAALL	
Response Variable		
Number of Response Levels	2	
Model	binary logit	
Optimization Technique	Fisher's scoring	

Number of Observations Read	85
Number of Observations Used	68

Response Profile		
Ordered Value	USGfirst24binarylowcutoff	Total Frequency
1	0	41
2	1	27

Probability modeled is USGfirst24binarylowcutoff=0.

Note: 17 observations were deleted due to missing values for the response or explanatory variables.

Model Convergence Status
Convergence criterion (GCONV=1E-8) satisfied.

Model Fit Statistics		
Criterion	Intercept Only	Intercept and Covariates
AIC	93.365	87.758
SC	95.584	107.733
-2 Log L	91.365	69.758

Testing Global Null Hypothesis: BETA=0			
Test	Chi-Square	DF	Pr > ChiSq
Likelihood Ratio	21.6071	8	0.0057
Score	17.5107	8	0.0252
Wald	13.0820	8	0.1091

Analysis of Maximum Likelihood Estimates					
Parameter	DF	Estimate	Standard Error	Wald Chi-Square	Pr > ChiSq
Intercept	1	-1.7063	1.5776	1.1697	0.2795
BWchange_2d_binary1p	1	0.4069	0.8417	0.2337	0.6288
Drink_water_morning	1	-0.9362	0.9150	1.0469	0.3062
Thirst_intensity_mor	1	-0.0173	0.0232	0.5583	0.4549
Thirst_distress_morn	1	-0.00384	0.00551	0.4844	0.4864
selfreport_fluidinta	1	0.00433	0.00441	0.9634	0.3263
Color_chart_Paper_mo	1	-0.4453	0.6922	0.4138	0.5200

The LOGISTIC Procedure

Analysis of Maximum Likelihood Estimates					
Parameter	DF	Estimate	Standard Error	Wald Chi-Square	Pr > ChiSq
Black1_seconds	1	0.0289	0.0242	1.4283	0.2320
selfreport_Urine_fre	1	0.3895	0.1555	6.2742	0.0123

Odds Ratio Estimates			
Effect	Point Estimate	95% Wald Confidence Limits	
BWchange_2d_binary1p	1.502	0.289	7.820
Drink_water_morning	0.392	0.065	2.356
Thirst_intensity_mor	0.983	0.939	1.028
Thirst_distress_morn	0.996	0.985	1.007
selfreport_fluidinta	1.004	0.996	1.013
Color_chart_Paper_mo	0.641	0.165	2.488
Black1_seconds	1.029	0.982	1.079
selfreport_Urine_fre	1.476	1.088	2.002

Association of Predicted Probabilities and Observed Responses			
Percent Concordant	79.4	Somers' D	0.588
Percent Discordant	20.6	Gamma	0.588
Percent Tied	0.0	Tau-a	0.286
Pairs	1107	c	0.794

MORNING MODEL likely to be self-reported variables (continuous) by participants (MORNING SR)

OPTION 2 (differentiating between color charts, 3D color chart) – continuous variables
BW change% based on two days; Drink water morning (yes/no); Thirst intensity morning (percentage); Thirst distress morning (percentage); Self-reported fluid intake first 24-hour (mL); Color chart 3D morning (score 1-7); Urine volume morning sample (seconds); Self-reported urine frequency over 24-hour.

The LOGISTIC Procedure

Model Information		
Data Set	WORK.FEMAALL	
Response Variable		
Number of Response Levels	2	
Model	binary logit	
Optimization Technique	Fisher's scoring	

Number of Observations Read	85
Number of Observations Used	68

Response Profile		
Ordered Value	USGfirst24binarylowcutoff	Total Frequency
1	0	41
2	1	27

Probability modeled is USGfirst24binarylowcutoff=0.

Note: 17 observations were deleted due to missing values for the response or explanatory variables.

Model Convergence Status
Convergence criterion (GCONV=1E-8) satisfied.

Model Fit Statistics		
Criterion	Intercept Only	Intercept and Covariates
AIC	93.365	87.600
SC	95.584	107.576
-2 Log L	91.365	69.600

Testing Global Null Hypothesis: BETA=0			
Test	Chi-Square	DF	Pr > ChiSq
Likelihood Ratio	21.7646	8	0.0054
Score	17.5263	8	0.0251
Wald	12.9165	8	0.1148

Analysis of Maximum Likelihood Estimates					
Parameter	DF	Estimate	Standard Error	Wald Chi-Square	Pr > ChiSq
Intercept	1	-1.3287	1.7910	0.5503	0.4582
BWchange_2d_binary1p	1	0.2706	0.8577	0.0996	0.7523
Drink_water_morning	1	-0.9137	0.9046	1.0202	0.3125
Thirst_intensity_mor	1	-0.0178	0.0231	0.5942	0.4408
Thirst_distress_morn	1	-0.00462	0.00576	0.6445	0.4221
selfreport_fluidinta	1	0.00419	0.00444	0.8934	0.3446
Color_chart_3D_morni	1	-0.1852	0.2454	0.5693	0.4505

The LOGISTIC Procedure

Analysis of Maximum Likelihood Estimates					
Parameter	DF	Estimate	Standard Error	Wald Chi-Square	Pr > ChiSq
Black1_seconds	1	0.0300	0.0230	1.7006	0.1922
selfreport_Urine_fre	1	0.4051	0.1609	6.3409	0.0118

Odds Ratio Estimates			
Effect	Point Estimate	95% Wald Confidence Limits	
BWchange_2d_binary1p	1.311	0.244	7.040
Drink_water_morning	0.401	0.068	2.361
Thirst_intensity_mor	0.982	0.939	1.028
Thirst_distress_morn	0.995	0.984	1.007
selfreport_fluidinta	1.004	0.996	1.013
Color_chart_3D_morni	0.831	0.514	1.344
Black1_seconds	1.030	0.985	1.078
selfreport_Urine_fre	1.499	1.094	2.055

Association of Predicted Probabilities and Observed Responses			
Percent Concordant	79.9	Somers' D	0.597
Percent Discordant	20.1	Gamma	0.597
Percent Tied	0.0	Tau-a	0.290
Pairs	1107	c	0.799

<u>MORNING MODEL measured continuous variables by the research team</u>
(MORNING ME)

OPTION 1 – measured continuous variables
Heart rate in the morning (bpm); Urine volume First 24-hour (mL); Urine volume morning sample (mL); Blood pressure, systolic (mmHg); Blood pressure, diastolic (mmHg); Total voids during the first 24-hour (count).

The LOGISTIC Procedure

Model Information		
Data Set	WORK.FEMAALL	
Response Variable		
Number of Response Levels	2	
Model	binary logit	
Optimization Technique	Fisher's scoring	

Number of Observations Read	85
Number of Observations Used	81

Response Profile		
Ordered Value	USGfirst24binarylowcutoff	Total Frequency
1	0	48
2	1	33

Probability modeled is USGfirst24binarylowcutoff=0.

Note: 4 observations were deleted due to missing values for the response or explanatory variables.

Model Convergence Status
Convergence criterion (GCONV=1E-8) satisfied.

Model Fit Statistics		
Criterion	Intercept Only	Intercept and Covariates
AIC	111.496	53.402
SC	113.890	70.163
-2 Log L	109.496	39.402

Testing Global Null Hypothesis: BETA=0			
Test	Chi-Square	DF	Pr > ChiSq
Likelihood Ratio	70.0940	6	<.0001
Score	36.4950	6	<.0001
Wald	15.0734	6	0.0197

Analysis of Maximum Likelihood Estimates					
Parameter	DF	Estimate	Standard Error	Wald Chi-Square	Pr > ChiSq
Intercept	1	-2.0556	4.3509	0.2232	0.6366
HR_morning	1	0.0155	0.0476	0.1059	0.7449
Urine_volume_First_2	1	0.00516	0.00142	13.2776	0.0003
Black1_volumeacc	1	-0.00347	0.00280	1.5352	0.2153
BPmorn_high	1	-0.1596	0.0544	8.6055	0.0034
BPmorn_low	1	0.1911	0.0765	6.2321	0.0125
Totalvoidfirst24hrac	1	-0.3684	0.2832	1.6928	0.1932

The LOGISTIC Procedure

Odds Ratio Estimates			
Effect	Point Estimate	95% Wald Confidence Limits	
HR_morning	1.016	0.925	1.115
Urine_volume_First_2	1.005	1.002	1.008
Black1_volumeacc	0.997	0.991	1.002
BPmorn_high	0.852	0.766	0.948
BPmorn_low	1.211	1.042	1.406
Totalvoidfirst24hrac	0.692	0.397	1.205

Association of Predicted Probabilities and Observed Responses			
Percent Concordant	96.8	Somers' D	0.937
Percent Discordant	3.2	Gamma	0.937
Percent Tied	0.0	Tau-a	0.458
Pairs	1584	c	0.968

MORNING MODEL likely to be self-reported binary (MORNING SR BIN)

OPTION 1 (differentiating between color charts, print color chart) – binary variables
BW change% based on two days (≤1%); Drink water morning (yes/no); Thirst intensity morning (≤40%); Thirst distress morning (≤40%); Self-reported fluid intake first 24-hour (sex-dependent, male ≥3.8 L, female ≥2.7 L); Color chart 3D morning (≤2); Self-reported urine frequency over 24-hour (≥7); Urine volume morning sample (≥16 seconds).

The LOGISTIC Procedure

Model Information		
Data Set	WORK.FEMAALL	
Response Variable		
Number of Response Levels	2	
Model	binary logit	
Optimization Technique	Fisher's scoring	

Number of Observations Read	85
Number of Observations Used	68

Response Profile		
Ordered Value	USGfirst24binarylowcutoff	Total Frequency
1	0	41
2	1	27

Probability modeled is USGfirst24binarylowcutoff=0.

Note: 17 observations were deleted due to missing values for the response or explanatory variables.

Model Convergence Status
Convergence criterion (GCONV=1E-8) satisfied.

Model Fit Statistics		
Criterion	Intercept Only	Intercept and Covariates
AIC	93.365	91.114
SC	95.584	111.090
-2 Log L	91.365	73.114

Testing Global Null Hypothesis: BETA=0			
Test	Chi-Square	DF	Pr > ChiSq
Likelihood Ratio	18.2507	8	0.0194
Score	16.1865	8	0.0398
Wald	12.4137	8	0.1337

Analysis of Maximum Likelihood Estimates					
Parameter	DF	Estimate	Standard Error	Wald Chi-Square	Pr > ChiSq
Intercept	1	-1.1030	1.1116	0.9847	0.3210
BWchange_2d_binary1p	1	0.7556	0.8536	0.7834	0.3761
Drink_water_morning	1	-1.2129	0.8498	2.0371	0.1535
Thirst_intensity_mor	1	-0.6569	0.7362	0.7961	0.3723
Thirst_distress_morn	1	1.5633	0.9718	2.5879	0.1077
Color_chart_Paper_mo	1	-0.2553	0.6699	0.1452	0.7032
selfreport_fluidinta	1	0.00663	0.00414	2.5625	0.1094

The LOGISTIC Procedure

Analysis of Maximum Likelihood Estimates					
Parameter	DF	Estimate	Standard Error	Wald Chi-Square	Pr > ChiSq
selfreport_Urine_fre	1	1.1179	0.6200	3.2515	0.0714
Black1_seconds_16s_c	1	1.5222	0.7716	3.8922	0.0485

Odds Ratio Estimates			
Effect	Point Estimate	95% Wald Confidence Limits	
BWchange_2d_binary1p	2.129	0.400	11.344
Drink_water_morning	0.297	0.056	1.573
Thirst_intensity_mor	0.518	0.122	2.195
Thirst_distress_morn	4.775	0.711	32.073
Color_chart_Paper_mo	0.775	0.208	2.880
selfreport_fluidinta	1.007	0.999	1.015
selfreport_Urine_fre	3.058	0.907	10.309
Black1_seconds_16s_c	4.582	1.010	20.788

Association of Predicted Probabilities and Observed Responses			
Percent Concordant	82.3	Somers' D	0.646
Percent Discordant	17.7	Gamma	0.646
Percent Tied	0.0	Tau-a	0.314
Pairs	1107	c	0.823

MORNING MODEL likely to be self-reported binary (MORNING SR BIN)

OPTION 2 (differentiating between color charts, 3D color chart) – binary variables
BW change% based on two days (≤1%); Drink water morning (yes/no); Thirst intensity morning (≤40%); Thirst distress morning (≤40%); Self-reported fluid intake first 24-hour (sex-dependent, male ≥3.8 L, female ≥2.7 L); Color chart 3D morning (≤2); Urine volume morning sample (≥16 seconds); Self-reported urine frequency over 24-hour (≥7).

The LOGISTIC Procedure

Model Information		
Data Set	WORK.FEMAALL	
Response Variable		
Number of Response Levels	2	
Model	binary logit	
Optimization Technique	Fisher's scoring	

Number of Observations Read	85
Number of Observations Used	68

Response Profile		
Ordered Value	USGfirst24binarylowcutoff	Total Frequency
1	0	41
2	1	27

Probability modeled is USGfirst24binarylowcutoff=0.

Note: 17 observations were deleted due to missing values for the response or explanatory variables.

Model Convergence Status
Convergence criterion (GCONV=1E-8) satisfied.

Model Fit Statistics		
Criterion	Intercept Only	Intercept and Covariates
AIC	93.365	91.255
SC	95.584	111.230
-2 Log L	91.365	73.255

Testing Global Null Hypothesis: BETA=0			
Test	Chi-Square	DF	Pr > ChiSq
Likelihood Ratio	18.1101	8	0.0204
Score	16.0868	8	0.0412
Wald	12.3339	8	0.1369

Analysis of Maximum Likelihood Estimates					
Parameter	DF	Estimate	Standard Error	Wald Chi-Square	Pr > ChiSq
Intercept	1	-1.2815	1.1344	1.2761	0.2586
BWchange_2d_binary1p	1	0.7342	0.8549	0.7375	0.3905
Drink_water_morning	1	-1.2125	0.8482	2.0433	0.1529
Thirst_intensity_mor	1	-0.6807	0.7436	0.8378	0.3600
Thirst_distress_morn	1	1.5593	0.9706	2.5808	0.1082
Color_chart_3D_morni	1	-0.0394	0.6600	0.0036	0.9524
selfreport_fluidinta	1	0.00679	0.00414	2.6937	0.1007

The LOGISTIC Procedure

Analysis of Maximum Likelihood Estimates					
Parameter	DF	Estimate	Standard Error	Wald Chi-Square	Pr > ChiSq
selfreport_Urine_fre	1	1.1282	0.6210	3.2999	0.0693
Black1_seconds_16s_c	1	1.6368	0.7662	4.5640	0.0327

Odds Ratio Estimates			
Effect	Point Estimate	95% Wald Confidence Limits	
BWchange_2d_binary1p	2.084	0.390	11.131
Drink_water_morning	0.297	0.056	1.568
Thirst_intensity_mor	0.506	0.118	2.175
Thirst_distress_morn	4.756	0.710	31.871
Color_chart_3D_morni	0.961	0.264	3.505
selfreport_fluidinta	1.007	0.999	1.015
selfreport_Urine_fre	3.090	0.915	10.438
Black1_seconds_16s_c	5.139	1.145	23.070

Association of Predicted Probabilities and Observed Responses			
Percent Concordant	82.9	Somers' D	0.659
Percent Discordant	17.1	Gamma	0.659
Percent Tied	0.0	Tau-a	0.320
Pairs	1107	c	0.829

MORNING MODEL measured binary
(MORNING ME BIN)

ONE OPTION ONLY

Total voids during the first 24-hour (≥7); Blood pressure, systolic (<120 mmHg); Blood pressure, diastolic (<80 mmHg); Urine volume First 24-hour (≥2000 mL); Urine volume morning sample (≥250 mL).

The LOGISTIC Procedure

Model Information		
Data Set	WORK.FEMAALL	
Response Variable		
Number of Response Levels	2	
Model	binary logit	
Optimization Technique	Fisher's scoring	

Number of Observations Read	85
Number of Observations Used	81

Response Profile		
Ordered Value	USGfirst24binarylowcutoff	Total Frequency
1	0	48
2	1	33

Probability modeled is USGfirst24binarylowcutoff=0.

Note: 4 observations were deleted due to missing values for the response or explanatory variables.

Model Convergence Status
Convergence criterion (GCONV=1E-8) satisfied.

Model Fit Statistics		
Criterion	Intercept Only	Intercept and Covariates
AIC	111.496	86.657
SC	113.890	98.629
-2 Log L	109.496	76.657

Testing Global Null Hypothesis: BETA=0			
Test	Chi-Square	DF	Pr > ChiSq
Likelihood Ratio	32.8394	4	<.0001
Score	30.9772	4	<.0001
Wald	25.3012	4	<.0001

Analysis of Maximum Likelihood Estimates					
Parameter	DF	Estimate	Standard Error	Wald Chi-Square	Pr > ChiSq
Intercept	1	-1.1376	0.8560	1.7662	0.1839
Totalvoidfirst24hr_7	1	-0.0959	0.7164	0.0179	0.8936
BP_binary_with_0_sma	1	-0.4327	0.6439	0.4515	0.5016
Urine_volume_First_2	1	2.9869	0.7221	17.1110	<.0001
Black1_volume_250mL_	1	0.2127	0.7538	0.0796	0.7778

The LOGISTIC Procedure

Odds Ratio Estimates			
Effect	Point Estimate	95% Wald Confidence Limits	
Totalvoidfirst24hr_7	0.909	0.223	3.700
BP_binary_with_0_sma	0.649	0.184	2.292
Urine_volume_First_2	19.825	4.815	81.631
Black1_volume_250mL_	1.237	0.282	5.420

Association of Predicted Probabilities and Observed Responses			
Percent Concordant	77.8	Somers' D	0.635
Percent Discordant	14.3	Gamma	0.690
Percent Tied	8.0	Tau-a	0.310
Pairs	1584	c	0.818

AFTERNOON MODEL likely to be self-reported variables (continuous) by participants (AFTERNOON SR)

OPTION 1 (differentiating between color charts, print color chart) – continuous variables
BW change% based on two days; Drink water afternoon (yes/no); Thirst intensity afternoon (percentage); Thirst distress afternoon (percentage); Self-reported fluid intake second 24-hour (mL); Color chart Paper afternoon (score 1-7); Urine volume afternoon sample (seconds); Self-reported urine frequency over 24-hour.

The LOGISTIC Procedure

Model Information		
Data Set	WORK.FEMAALL	
Response Variable		
Number of Response Levels	2	
Model	binary logit	
Optimization Technique	Fisher's scoring	

Number of Observations Read	85
Number of Observations Used	50

Response Profile		
Ordered Value	USGfirst24binarylowcutoff	Total Frequency
1	0	28
2	1	22

Probability modeled is USGfirst24binarylowcutoff=0.

Note: 35 observations were deleted due to missing values for the response or explanatory variables.

Model Convergence Status
Convergence criterion (GCONV=1E-8) satisfied.

Model Fit Statistics		
Criterion	Intercept Only	Intercept and Covariates
AIC	70.593	77.342
SC	72.505	94.550
-2 Log L	68.593	59.342

Testing Global Null Hypothesis: BETA=0			
Test	Chi-Square	DF	Pr > ChiSq
Likelihood Ratio	9.2508	8	0.3216
Score	8.4177	8	0.3938
Wald	7.0627	8	0.5299

Analysis of Maximum Likelihood Estimates					
Parameter	DF	Estimate	Standard Error	Wald Chi-Square	Pr > ChiSq
Intercept	1	-3.1026	2.0879	2.2081	0.1373
BWchange_2d_binary1p	1	1.6583	1.3220	1.5734	0.2097
Drink_water_afternoo	1	0.7140	0.7529	0.8994	0.3429
SELFThirst_distress_	1	-0.0356	0.0466	0.5824	0.4454
SELFThirst_intensity	1	-0.00025	0.0436	0.0000	0.9955
selfreport_fluidinta	1	0.00518	0.00552	0.8818	0.3477
Totalvoid_2nd24hr	1	0.1580	0.1656	0.9104	0.3400

The LOGISTIC Procedure

Analysis of Maximum Likelihood Estimates					
Parameter	DF	Estimate	Standard Error	Wald Chi-Square	Pr > ChiSq
UC_Paper_afternoon	1	0.2100	0.3529	0.3540	0.5518
Black2_secondsacc	1	0.0627	0.0455	1.9001	0.1681

Odds Ratio Estimates			
Effect	Point Estimate	95% Wald Confidence Limits	
BWchange_2d_binary1p	5.250	0.393	70.063
Drink_water_afternoo	2.042	0.467	8.933
SELFThirst_distress_	0.965	0.881	1.057
SELFThirst_intensity	1.000	0.918	1.089
selfreport_fluidinta	1.005	0.994	1.016
Totalvoid_2nd24hr	1.171	0.847	1.620
UC_Paper_afternoon	1.234	0.618	2.463
Black2_secondsacc	1.065	0.974	1.164

Association of Predicted Probabilities and Observed Responses			
Percent Concordant	73.9	Somers' D	0.477
Percent Discordant	26.1	Gamma	0.477
Percent Tied	0.0	Tau-a	0.240
Pairs	616	c	0.739

<u>AFTERNOON MODEL likely to be self-reported variables (continuous) by participants</u> (AFTERNOON SR)

OPTION 2 (differentiating between color charts, 3D color chart) – continuous variables
BW change% based on two days; Drink water afternoon (yes/no); Thirst intensity afternoon (percentage); Thirst distress afternoon (percentage); Self-reported fluid intake second 24-hour (mL); Color chart 3D afternoon (score 1-7); Urine volume afternoon sample (seconds); Self-reported urine frequency over 24-hour.

The LOGISTIC Procedure

Model Information		
Data Set	WORK.FEMAALL	
Response Variable		
Number of Response Levels	2	
Model	binary logit	
Optimization Technique	Fisher's scoring	

Number of Observations Read	85
Number of Observations Used	50

Response Profile		
Ordered Value	USGfirst24binarylowcutoff	Total Frequency
1	0	28
2	1	22

Probability modeled is USGfirst24binarylowcutoff=0.

Note: 35 observations were deleted due to missing values for the response or explanatory variables.

Model Convergence Status
Convergence criterion (GCONV=1E-8) satisfied.

Model Fit Statistics		
Criterion	Intercept Only	Intercept and Covariates
AIC	70.593	77.520
SC	72.505	94.728
-2 Log L	68.593	59.520

Testing Global Null Hypothesis: BETA=0			
Test	Chi-Square	DF	Pr > ChiSq
Likelihood Ratio	9.0732	8	0.3362
Score	8.2316	8	0.4112
Wald	6.8945	8	0.5481

Analysis of Maximum Likelihood Estimates					
Parameter	DF	Estimate	Standard Error	Wald Chi-Square	Pr > ChiSq
Intercept	1	-1.5641	1.9366	0.6523	0.4193
BWchange_2d_binary1p	1	1.6908	1.3335	1.6078	0.2048
Drink_water_afternoo	1	0.5992	0.7641	0.6150	0.4329
SELFThirst_distress_	1	-0.0366	0.0461	0.6303	0.4273
SELFThirst_intensity	1	0.00512	0.0436	0.0138	0.9066
selfreport_fluidinta	1	0.00430	0.00539	0.6361	0.4251
Totalvoid_2nd24hr	1	0.1024	0.1635	0.3923	0.5311

The LOGISTIC Procedure

Analysis of Maximum Likelihood Estimates					
Parameter	DF	Estimate	Standard Error	Wald Chi-Square	Pr > ChiSq
UC_3D_afternoon	1	-0.1390	0.3241	0.1840	0.6680
Black2_secondsacc	1	0.0433	0.0427	1.0297	0.3102

Odds Ratio Estimates			
Effect	Point Estimate	95% Wald Confidence Limits	
BWchange_2d_binary1p	5.424	0.397	74.025
Drink_water_afternoo	1.821	0.407	8.141
SELFThirst_distress_	0.964	0.881	1.055
SELFThirst_intensity	1.005	0.923	1.095
selfreport_fluidinta	1.004	0.994	1.015
Totalvoid_2nd24hr	1.108	0.804	1.526
UC_3D_afternoon	0.870	0.461	1.642
Black2_secondsacc	1.044	0.960	1.135

Association of Predicted Probabilities and Observed Responses			
Percent Concordant	74.2	Somers' D	0.484
Percent Discordant	25.8	Gamma	0.484
Percent Tied	0.0	Tau-a	0.243
Pairs	616	c	0.742

<u>AFTERNOON MODEL measured continuous variables by the research team</u>
(AFTERNOON ME)

OPTION 1 – measured continuous variables
Heart rate in the afternoon (bpm); Urine volume Second 24-hour (mL); Urine volume afternoon sample (mL); Blood pressure, systolic (mmHg); Blood pressure, diastolic (mmHg); Total voids during the second 24-hour (count).

The LOGISTIC Procedure

Model Information		
Data Set	WORK.FEMAALL	
Response Variable		
Number of Response Levels	2	
Model	binary logit	
Optimization Technique	Fisher's scoring	

Number of Observations Read	85
Number of Observations Used	78

Response Profile		
Ordered Value	USGfirst24binarylowcutoff	Total Frequency
1	0	46
2	1	32

Probability modeled is USGfirst24binarylowcutoff=0.

Note: 7 observations were deleted due to missing values for the response or explanatory variables.

Model Convergence Status
Convergence criterion (GCONV=1E-8) satisfied.

Model Fit Statistics		
Criterion	Intercept Only	Intercept and Covariates
AIC	107.604	68.674
SC	109.961	85.171
-2 Log L	105.604	54.674

Testing Global Null Hypothesis: BETA=0			
Test	Chi-Square	DF	Pr > ChiSq
Likelihood Ratio	50.9309	6	<.0001
Score	29.8854	6	<.0001
Wald	16.6251	6	0.0108

Analysis of Maximum Likelihood Estimates					
Parameter	DF	Estimate	Standard Error	Wald Chi-Square	Pr > ChiSq
Intercept	1	0.1259	4.2867	0.0009	0.9766
HR_afternoon	1	-0.0367	0.0348	1.1104	0.2920
Urine_volume_First_2	1	0.00299	0.000753	15.7705	<.0001
Black2_Volumeacc	1	-0.00114	0.00179	0.4039	0.5251
BPafternoon_high	1	-0.0244	0.0271	0.8097	0.3682
BPafternoon_low	1	0.0196	0.0510	0.1468	0.7016
Totalvoidsecond24hr	1	-0.2117	0.1771	1.4286	0.2320

The LOGISTIC Procedure

Odds Ratio Estimates			
Effect	Point Estimate	95% Wald Confidence Limits	
HR_afternoon	0.964	0.900	1.032
Urine_volume_First_2	1.003	1.002	1.004
Black2_Volumeacc	0.999	0.995	1.002
BPafternoon_high	0.976	0.926	1.029
BPafternoon_low	1.020	0.923	1.127
Totalvoidsecond24hr	0.809	0.572	1.145

Association of Predicted Probabilities and Observed Responses			
Percent Concordant	92.9	Somers' D	0.857
Percent Discordant	7.1	Gamma	0.857
Percent Tied	0.0	Tau-a	0.420
Pairs	1472	c	0.929

<u>AFTERNOON MODEL likely to be self-reported binary</u>
(AFTERNOON SR BIN)

OPTION 1 (differentiating between color charts, print color chart) – binary variables
BW change% based on two days (≤1%); Drink water afternoon (yes/no); Thirst intensity afternoon (≤40%); Thirst distress afternoon (≤40%); Self-reported fluid intake second 24-hour (sex-dependent, male ≥3.8 L, female ≥2.7 L); Color chart 3D afternoon (≤2); Urine volume afternoon sample (≥16 seconds); Self-reported urine frequency over 24-hour (≥7).

The LOGISTIC Procedure

Model Information		
Data Set	WORK.FEMAALL	
Response Variable		
Number of Response Levels	2	
Model	binary logit	
Optimization Technique	Fisher's scoring	

Number of Observations Read	85
Number of Observations Used	40

Response Profile		
Ordered Value	USGfirst24binarylowcutoff	Total Frequency
1	0	22
2	1	18

Probability modeled is USGfirst24binarylowcutoff=0.

Note: 45 observations were deleted due to missing values for the response or explanatory variables.

Model Convergence Status
Convergence criterion (GCONV=1E-8) satisfied.

Model Fit Statistics		
Criterion	Intercept Only	Intercept and Covariates
AIC	57.051	63.947
SC	58.740	79.147
-2 Log L	55.051	45.947

Testing Global Null Hypothesis: BETA=0			
Test	Chi-Square	DF	Pr > ChiSq
Likelihood Ratio	9.1037	8	0.3336
Score	8.1937	8	0.4148
Wald	6.5974	8	0.5806

Analysis of Maximum Likelihood Estimates					
Parameter	DF	Estimate	Standard Error	Wald Chi-Square	Pr > ChiSq
Intercept	1	-3.3870	1.7925	3.5704	0.0588
BWchange_2d_binary1p	1	1.2112	1.3947	0.7541	0.3852
Drink_water_afternoo	1	0.5188	0.8042	0.4161	0.5189
SELFThirst_distress_	1	-0.9795	1.6344	0.3592	0.5490
SELFThirst_intensity	1	0.5352	1.1591	0.2132	0.6443
UC_Paper_afternoon_b	1	0.1497	0.9418	0.0253	0.8737
selfreport_fluidinta	1	0.00930	0.00537	3.0006	0.0832

The LOGISTIC Procedure

Analysis of Maximum Likelihood Estimates					
Parameter	DF	Estimate	Standard Error	Wald Chi-Square	Pr > ChiSq
selfreport_Urine_fre	1	0.1910	0.1846	1.0699	0.3010
Black2_seconds_16s_c	1	1.0468	0.8484	1.5223	0.2173

Odds Ratio Estimates			
Effect	Point Estimate	95% Wald Confidence Limits	
BWchange_2d_binary1p	3.357	0.218	51.665
Drink_water_afternoo	1.680	0.347	8.126
SELFThirst_distress_	0.375	0.015	9.242
SELFThirst_intensity	1.708	0.176	16.561
UC_Paper_afternoon_b	1.161	0.183	7.356
selfreport_fluidinta	1.009	0.999	1.020
selfreport_Urine_fre	1.210	0.843	1.738
Black2_seconds_16s_c	2.849	0.540	15.026

Association of Predicted Probabilities and Observed Responses			
Percent Concordant	76.8	Somers' D	0.535
Percent Discordant	23.2	Gamma	0.535
Percent Tied	0.0	Tau-a	0.272
Pairs	396	c	0.768

AFTERNOON MODEL likely to be self-reported binary
(AFTERNOON SR BIN)

OPTION 2 (differentiating between color charts, 3D color chart) – binary variables
BW change% based on two days (≤1%); Drink water afternoon (yes/no); Thirst intensity afternoon (≤40%); Thirst distress afternoon (≤40%); Self-reported fluid intake second 24-hour (sex-dependent, male ≥3.8 L, female ≥2.7 L); Color chart 3D afternoon (≤2); Urine volume afternoon sample (≥16 seconds); Self-reported urine frequency over 24-hour (≥7).

The LOGISTIC Procedure

Model Information	
Data Set	WORK.FEMAALL
Response Variable	
Number of Response Levels	2
Model	binary logit
Optimization Technique	Fisher's scoring

Number of Observations Read	85
Number of Observations Used	40

Response Profile		
Ordered Value	USGfirst24binarylowcutoff	Total Frequency
1	0	22
2	1	18

Probability modeled is USGfirst24binarylowcutoff=0.

Note: 45 observations were deleted due to missing values for the response or explanatory variables.

Model Convergence Status
Convergence criterion (GCONV=1E-8) satisfied.

Model Fit Statistics		
Criterion	Intercept Only	Intercept and Covariates
AIC	57.051	62.387
SC	58.740	77.587
-2 Log L	55.051	44.387

Testing Global Null Hypothesis: BETA=0			
Test	Chi-Square	DF	Pr > ChiSq
Likelihood Ratio	10.6642	8	0.2215
Score	9.5667	8	0.2968
Wald	7.5973	8	0.4738

Analysis of Maximum Likelihood Estimates					
Parameter	DF	Estimate	Standard Error	Wald Chi-Square	Pr > ChiSq
Intercept	1	-2.3206	1.5878	2.1360	0.1439
BWchange_2d_binary1p	1	1.4108	1.5674	0.8102	0.3681
Drink_water_afternoo	1	0.3693	0.8061	0.2099	0.6469
SELFThirst_distress_	1	-1.5656	1.7338	0.8154	0.3665
SELFThirst_intensity	1	0.9310	1.1977	0.6042	0.4370
UC_3D_afternoon_bina	1	-1.1176	0.9100	1.5082	0.2194
selfreport_fluidinta	1	0.00988	0.00566	3.0505	0.0807

The LOGISTIC Procedure

Analysis of Maximum Likelihood Estimates					
Parameter	DF	Estimate	Standard Error	Wald Chi-Square	Pr > ChiSq
selfreport_Urine_fre	1	0.1017	0.1708	0.3549	0.5514
Black2_seconds_16s_c	1	0.7958	0.8333	0.9121	0.3396

Odds Ratio Estimates			
Effect	Point Estimate	95% Wald Confidence Limits	
BWchange_2d_binary1p	4.099	0.190	88.494
Drink_water_afternoo	1.447	0.298	7.023
SELFThirst_distress_	0.209	0.007	6.250
SELFThirst_intensity	2.537	0.243	26.534
UC_3D_afternoon_bina	0.327	0.055	1.947
selfreport_fluidinta	1.010	0.999	1.021
selfreport_Urine_fre	1.107	0.792	1.547
Black2_seconds_16s_c	2.216	0.433	11.348

Association of Predicted Probabilities and Observed Responses			
Percent Concordant	80.6	Somers' D	0.611
Percent Discordant	19.4	Gamma	0.611
Percent Tied	0.0	Tau-a	0.310
Pairs	396	c	0.806

AFTERNOON MODEL measured binary
(AFTERNOON ME BIN)

ONE OPTION ONLY

Total voids during the second 24-hour (≥7); Blood pressure, systolic (<120 mmHg); Blood pressure, diastolic (<80 mmHg); Urine volume Second 24-hour (≥2000 mL); Urine volume afternoon sample (≥250 mL);

The LOGISTIC Procedure

Model Information		
Data Set	WORK.FEMAALL	
Response Variable		
Number of Response Levels	2	
Model	binary logit	
Optimization Technique	Fisher's scoring	

Number of Observations Read	85
Number of Observations Used	78

Response Profile		
Ordered Value	USGfirst24binarylowcutoff	Total Frequency
1	0	47
2	1	31

Probability modeled is USGfirst24binarylowcutoff=0.

Note: 7 observations were deleted due to missing values for the response or explanatory variables.

Model Convergence Status
Convergence criterion (GCONV=1E-8) satisfied.

Model Fit Statistics		
Criterion	Intercept Only	Intercept and Covariates
AIC	106.825	103.675
SC	109.182	115.459
-2 Log L	104.825	93.675

Testing Global Null Hypothesis: BETA=0			
Test	Chi-Square	DF	Pr > ChiSq
Likelihood Ratio	11.1502	4	0.0249
Score	10.8107	4	0.0288
Wald	9.8431	4	0.0432

Analysis of Maximum Likelihood Estimates					
Parameter	DF	Estimate	Standard Error	Wald Chi-Square	Pr > ChiSq
Intercept	1	-0.1339	0.5341	0.0629	0.8020
Totalvoidsecond24hr_	1	0.1384	0.5781	0.0573	0.8108
BP_afternoon_Ratio_b	1	-0.7078	0.5414	1.7089	0.1911
Urine_volume_Second_	1	1.1695	0.6195	3.5637	0.0591
Black2_volume_250mL_	1	0.5430	0.5606	0.9384	0.3327

The LOGISTIC Procedure

Odds Ratio Estimates			
Effect	Point Estimate	95% Wald Confidence Limits	
Totalvoidsecond24hr_	1.148	0.370	3.566
BP_afternoon_Ratio_b	0.493	0.171	1.424
Urine_volume_Second_	3.220	0.956	10.844
Black2_volume_250mL_	1.721	0.574	5.164

Association of Predicted Probabilities and Observed Responses			
Percent Concordant	66.2	Somers' D	0.423
Percent Discordant	23.9	Gamma	0.470
Percent Tied	10.0	Tau-a	0.205
Pairs	1457	c	0.711

The LOGISTIC Procedure

Model Information		
Data Set	WORK.FEMAALL	
Response Variable		
Number of Response Levels	2	
Model	binary logit	
Optimization Technique	Fisher's scoring	

Number of Observations Read	85
Number of Observations Used	79

Response Profile		
Ordered Value	USGfirst24binarylowcutoff	Total Frequency
1	0	47
2	1	32

Probability modeled is USGfirst24binarylowcutoff=0.

Note: 6 observations were deleted due to missing values for the response or explanatory variables.

Model Convergence Status
Convergence criterion (GCONV=1E-8) satisfied.

Model Fit Statistics		
Criterion	Intercept Only	Intercept and Covariates
AIC	108.652	100.879
SC	111.021	105.618
-2 Log L	106.652	96.879

Testing Global Null Hypothesis: BETA=0			
Test	Chi-Square	DF	Pr > ChiSq
Likelihood Ratio	9.7731	1	0.0018
Score	9.7897	1	0.0018
Wald	9.1040	1	0.0026

Analysis of Maximum Likelihood Estimates					
Parameter	DF	Estimate	Standard Error	Wald Chi-Square	Pr > ChiSq
Intercept	1	0.8910	0.2969	9.0064	0.0027
UC_3D_afternoon_bina	1	-1.5841	0.5250	9.1040	0.0026

Odds Ratio Estimates			
Effect	Point Estimate	95% Wald Confidence Limits	
UC_3D_afternoon_bina	0.205	0.073	0.574

The LOGISTIC Procedure

Association of Predicted Probabilities and Observed Responses			
Percent Concordant	41.5	Somers' D	0.330
Percent Discordant	8.5	Gamma	0.660
Percent Tied	50.0	Tau-a	0.161
Pairs	1504	c	0.665
