# Supplementary material for: The accuracy of a 4-item hydration self-assessment model to classify urine concentration using different cut-offs
Source: Perform Nutr. 2026 Apr 7;2(1):8. doi: 10.1186/s44410-026-00024-y (PMC13056763; doi:10.1186/s44410-026-00024-y)
Supplement: Supplementary file 2 — Supplementary Material 2. [file 44410_2026_24_MOESM2_ESM.pdf]

Supplementary File 2

Modeling to determine the Area Under the Curve (AUC) for 4-item model and the individual variables for all n=85 participants (10 models). In which "c" is the AUC.

This file represents 10 models for the morning assessment (5 for low and 5 for high USG cut-off), and 10 models for the afternoon assessment for USG (5 for low and 5 for high USG cut-off).

Morning modeling was performed against the 1st 24h USG, and the afternoon modeling was performed against the 2nd 24-hour USG (≤1.012 and ≥1.020) categorized as low vs. higher.

Table of contents

4-ITEM MODEL Morning vs. USG (Low cut-off)

Self-reported fluid intake MODEL Morning vs. USG (Low cut-off)

Self-reported urine frequency MODEL Morning vs. USG (Low cut-off)

Self-reported first morning urine volume MODEL Moring vs. USG (Low cut-off)

Self-reported 3D urine color chary MODEL Morning vs. USG (Low cut-off)

4-ITEM MODEL Afternoon vs. USG (Low cut-off)

Self-reported fluid intake MODEL Afternoon vs. USG (Low cut-off)

Self-reported urine frequency MODEL Afternoon vs. USG (Low cut-off)

Self-reported first Afternoon urine volume MODEL Moring vs. USG (Low cut-off)

Self-reported 3D urine color chary MODEL Afternoon vs. USG (Low cut-off)

4-ITEM MODEL Morning vs. USG (High cutoff)

Self-reported fluid intake MODEL Morning vs. USG (High cutoff)

Self-reported urine frequency MODEL Morning vs. USG (High cutoff)

Self-reported first morning urine volume MODEL Moring vs. USG (High cutoff)

Self-reported 3D urine color chary MODEL Morning vs. USG (High cutoff)

4-ITEM MODEL Afternoon vs. USG (High cutoff)

Self-reported fluid intake MODEL Afternoon vs. USG (High cutoff)

Self-reported urine frequency MODEL Afternoon vs. USG (High cutoff)

Self-reported first Afternoon urine volume MODEL Moring vs. USG (High cutoff)

Self-reported 3D urine color chary MODEL Afternoon vs. USG (High cutoff)

4-ITEM MODEL Morning vs. USG (Low cut-off)

The LOGISTIC Procedure

Model Information		
Data Set	WORK.FEMAALL	
Response Variable		
Number of Response Levels	2	
Model	binary logit	
Optimization Technique	Fisher's scoring	

Number of Observations Read	85
Number of Observations Used	80

Response Profile		
Ordered Value	USGfirst24binarylowcutoff	Total Frequency
1	0	48
2	1	32

Probability modeled is USGfirst24binarylowcutoff=0.

Note: 5 observations were deleted due to missing values for the response or explanatory variables.

Model Convergence Status
Convergence criterion (GCONV=1E-8) satisfied.

Model Fit Statistics		
Criterion	Intercept Only	Intercept and Covariates
AIC	109.682	105.710
SC	112.064	117.620
-2 Log L	107.682	95.710

Testing Global Null Hypothesis: BETA=0			
Test	Chi-Square	DF	Pr > ChiSq
Likelihood Ratio	11.9721	4	0.0176
Score	11.0951	4	0.0255
Wald	9.7369	4	0.0451

Analysis of Maximum Likelihood Estimates					
Parameter	DF	Estimate	Standard Error	Wald Chi-Square	Pr > ChiSq
Intercept	1	-1.2250	0.9110	1.8079	0.1788
selfreport_fluidinta	1	0.00561	0.00333	2.8392	0.0920
selfreport_Urine_fre	1	1.1146	0.5129	4.7228	0.0298
Black1_volume_250mL_	1	0.5628	0.6867	0.6715	0.4125
Color_chart_3D_morni	1	-0.4575	0.5336	0.7352	0.3912

The LOGISTIC Procedure

Odds Ratio Estimates			
Effect	Point Estimate	95% Wald Confidence Limits	
selfreport_fluidinta	1.006	0.999	1.012
selfreport_Urine_fre	3.048	1.116	8.329
Black1_volume_250mL_	1.756	0.457	6.745
Color_chart_3D_morni	0.633	0.222	1.801

Association of Predicted Probabilities and Observed Responses			
Percent Concordant	71.5	Somers' D	0.434
Percent Discordant	28.2	Gamma	0.435
Percent Tied	0.3	Tau-a	0.211
Pairs	1536	c	0.717

Self-reported fluid intake MODEL Morning vs. USG (Low cut-off)

The LOGISTIC Procedure

Model Information		
Data Set	WORK.FEMAALL	
Response Variable		
Number of Response Levels	2	
Model	binary logit	
Optimization Technique	Fisher's scoring	

Number of Observations Read	85
Number of Observations Used	80

Response Profile		
Ordered Value	USGfirst24binarylowcutoff	Total Frequency
1	0	48
2	1	32

Probability modeled is USGfirst24binarylowcutoff=0.

Note: 5 observations were deleted due to missing values for the response or explanatory variables.

Model Convergence Status
Convergence criterion (GCONV=1E-8) satisfied.

Model Fit Statistics		
Criterion	Intercept Only	Intercept and Covariates
AIC	109.682	105.927
SC	112.064	110.691
-2 Log L	107.682	101.927

Testing Global Null Hypothesis: BETA=0			
Test	Chi-Square	DF	Pr > ChiSq
Likelihood Ratio	5.7553	1	0.0164
Score	5.2921	1	0.0214
Wald	4.8684	1	0.0274

Analysis of Maximum Likelihood Estimates					
Parameter	DF	Estimate	Standard Error	Wald Chi-Square	Pr > ChiSq
Intercept	1	-0.5557	0.4783	1.3497	0.2453
selfreport_fluidinta	1	0.00699	0.00317	4.8684	0.0274

Odds Ratio Estimates			
Effect	Point Estimate	95% Wald Confidence Limits	
selfreport_fluidinta	1.007	1.001	1.013

The LOGISTIC Procedure

Association of Predicted Probabilities and Observed Responses			
Percent Concordant	63.2	Somers' D	0.279
Percent Discordant	35.3	Gamma	0.283
Percent Tied	1.6	Tau-a	0.135
Pairs	1536	c	0.639

Self-reported urine frequency MODEL Morning vs. USG (Low cut-off)

The LOGISTIC Procedure

Model Information		
Data Set	WORK.FEMAALL	
Response Variable		
Number of Response Levels	2	
Model	binary logit	
Optimization Technique	Fisher's scoring	

Number of Observations Read	85
Number of Observations Used	81

Response Profile		
Ordered Value	USGfirst24binarylowcutoff	Total Frequency
1	0	48
2	1	33

Probability modeled is USGfirst24binarylowcutoff=0.

Note: 4 observations were deleted due to missing values for the response or explanatory variables.

Model Convergence Status
Convergence criterion (GCONV=1E-8) satisfied.

Model Fit Statistics		
Criterion	Intercept Only	Intercept and Covariates
AIC	111.496	106.580
SC	113.890	111.369
-2 Log L	109.496	102.580

Testing Global Null Hypothesis: BETA=0			
Test	Chi-Square	DF	Pr > ChiSq
Likelihood Ratio	6.9156	1	0.0085
Score	6.8677	1	0.0088
Wald	6.6395	1	0.0100

Analysis of Maximum Likelihood Estimates					
Parameter	DF	Estimate	Standard Error	Wald Chi-Square	Pr > ChiSq
Intercept	1	-0.2877	0.3416	0.7094	0.3996
selfreport_Urine_fre	1	1.2192	0.4732	6.6395	0.0100

Odds Ratio Estimates			
Effect	Point Estimate	95% Wald Confidence Limits	
selfreport_Urine_fre	3.385	1.339	8.556

The LOGISTIC Procedure

Association of Predicted Probabilities and Observed Responses			
Percent Concordant	41.7	Somers' D	0.294
Percent Discordant	12.3	Gamma	0.544
Percent Tied	46.0	Tau-a	0.144
Pairs	1584	c	0.647

Self-reported first morning urine volume MODEL Moring vs. USG (Low cut-off)

The LOGISTIC Procedure

Model Information		
**Data Set**	WORK.FEMAALL	
**Response Variable**	[illegible]	[illegible]
**Number of Response Levels**	2	
**Model**	binary logit	
**Optimization Technique**	Fisher's scoring	

**Number of Observations Read**	85
**Number of Observations Used**	81

Response Profile		
**Ordered Value**	**USGfirst24binarylowcutoff**	**Total Frequency**
**1**	0	48
**2**	1	33

Probability modeled is USGfirst24binarylowcutoff=0.

Note: 4 observations were deleted due to missing values for the response or explanatory variables.

Model Convergence Status
Convergence criterion (GCONV=1E-8) satisfied.

Model Fit Statistics		
**Criterion**	**Intercept Only**	**Intercept and Covariates**
**AIC**	111.496	112.903
**SC**	113.890	117.692
**-2 Log L**	109.496	108.903

Testing Global Null Hypothesis: BETA=0			
**Test**	**Chi-Square**	**DF**	**Pr > ChiSq**
**Likelihood Ratio**	0.5930	1	0.4413
**Score**	0.6010	1	0.4382
**Wald**	0.5952	1	0.4404

Analysis of Maximum Likelihood Estimates					
**Parameter**	**DF**	**Estimate**	**Standard Error**	**Wald Chi-Square**	**Pr > ChiSq**
**Intercept**	1	1.147E-7	0.5345	0.0000	1.0000
**Black1_volume_250mL_**	1	0.4555	0.5904	0.5952	0.4404

Odds Ratio Estimates			
**Effect**	**Point Estimate**	**95% Wald Confidence Limits**	
**Black1_volume_250mL_**	1.577	0.496	5.016

The LOGISTIC Procedure

Association of Predicted Probabilities and Observed Responses			
**Percent Concordant**	18.1	**Somers' D**	0.066
**Percent Discordant**	11.5	**Gamma**	0.224
**Percent Tied**	70.4	**Tau-a**	0.032
**Pairs**	1584	**c**	0.533

Self-reported 3D urine color chary MODEL Morning vs. USG (Low cut-off)

The LOGISTIC Procedure

Model Information		
Data Set	WORK.FEMAALL	
Response Variable		
Number of Response Levels	2	
Model	binary logit	
Optimization Technique	Fisher's scoring	

Number of Observations Read	85
Number of Observations Used	81

Response Profile		
Ordered Value	USGfirst24binarylowcutoff	Total Frequency
1	0	48
2	1	33

Probability modeled is USGfirst24binarylowcutoff=0.

Note: 4 observations were deleted due to missing values for the response or explanatory variables.

Model Convergence Status
Convergence criterion (GCONV=1E-8) satisfied.

Model Fit Statistics		
Criterion	Intercept Only	Intercept and Covariates
AIC	111.496	111.730
SC	113.890	116.519
-2 Log L	109.496	107.730

Testing Global Null Hypothesis: BETA=0			
Test	Chi-Square	DF	Pr > ChiSq
Likelihood Ratio	1.7661	1	0.1839
Score	1.7643	1	0.1841
Wald	1.7495	1	0.1859

Analysis of Maximum Likelihood Estimates					
Parameter	DF	Estimate	Standard Error	Wald Chi-Square	Pr > ChiSq
Intercept	1	0.6592	0.3180	4.2965	0.0382
Color_chart_3D_morni	1	-0.6052	0.4575	1.7495	0.1859

Odds Ratio Estimates			
Effect	Point Estimate	95% Wald Confidence Limits	
Color_chart_3D_morni	0.546	0.223	1.339

The LOGISTIC Procedure

Association of Predicted Probabilities and Observed Responses			
Percent Concordant	33.0	Somers' D	0.150
Percent Discordant	18.0	Gamma	0.294
Percent Tied	49.1	Tau-a	0.073
Pairs	1584	c	0.575

4-ITEM MODEL Afternoon vs. USG (Low cut-off)

The LOGISTIC Procedure

Model Information	
Data Set	WORK.FEMAALL
Response Variable	
Number of Response Levels	2
Model	binary logit
Optimization Technique	Fisher's scoring

Number of Observations Read	85
Number of Observations Used	67

Response Profile		
Ordered Value	USG_2nd_binary_low	Total Frequency
1	0	38
2	1	29

Probability modeled is USG_2nd_binary_low='0'.

Note: 18 observations were deleted due to missing values for the response or explanatory variables.

Model Convergence Status
Convergence criterion (GCONV=1E-8) satisfied.

Model Fit Statistics		
Criterion	Intercept Only	Intercept and Covariates
AIC	93.669	74.197
SC	95.874	85.220
-2 Log L	91.669	64.197

The LOGISTIC Procedure

Testing Global Null Hypothesis: BETA=0			
Test	Chi-Square	DF	Pr > ChiSq
Likelihood Ratio	27.4722	4	<.0001
Score	24.1465	4	<.0001
Wald	17.5160	4	0.0015

Analysis of Maximum Likelihood Estimates					
Parameter	DF	Estimate	Standard Error	Wald Chi-Square	Pr > ChiSq
Intercept	1	-1.7030	0.8471	4.0415	0.0444
selfreport_fluidinta	1	1.9151	0.6535	8.5874	0.0034
missurinefreq	1	1.0804	0.6424	2.8281	0.0926
Black2_volume_250mL_	1	1.1294	0.6924	2.6604	0.1029
UC_3D_afternoon_bina	1	-1.2724	0.7276	3.0580	0.0803

selfreport_fluidinta	6.787	1.886	24.432
missurinefreq	2.946	0.836	10.377
Black2_volume_250mL_	3.094	0.796	12.020
UC_3D_afternoon_bina	0.280	0.067	1.166

Association of Predicted Probabilities and Observed Responses			
Percent Concordant	82.4	Somers' D	0.693
Percent Discordant	13.1	Gamma	0.726
Percent Tied	4.5	Tau-a	0.346
Pairs	1102	c	0.847

Self-reported fluid intake MODEL Afternoon vs. USG (Low cut-off)

The LOGISTIC Procedure

Model Information	
Data Set	WORK.FEMAALL
Response Variable	
Number of Response Levels	2
Model	binary logit
Optimization Technique	Fisher's scoring

Number of Observations Read	85
Number of Observations Used	67

Response Profile		
Ordered Value	USG_2nd_binary_low	Total Frequency
1	0	38
2	1	29

Probability modeled is USG_2nd_binary_low='0'.

Note: 18 observations were deleted due to missing values for the response or explanatory variables.

Model Convergence Status
Convergence criterion (GCONV=1E-8) satisfied.

Model Fit Statistics		
Criterion	Intercept Only	Intercept and Covariates
AIC	93.669	81.966
SC	95.874	86.376
-2 Log L	91.669	77.966

Testing Global Null Hypothesis: BETA=0			
Test	Chi-Square	DF	Pr > ChiSq
Likelihood Ratio	13.7029	1	0.0002
Score	13.3843	1	0.0003
Wald	12.0257	1	0.0005

Analysis of Maximum Likelihood Estimates					
Parameter	DF	Estimate	Standard Error	Wald Chi-Square	Pr > ChiSq
Intercept	1	-1.0415	0.4749	4.8101	0.0283
selfreport_fluidinta	1	2.0223	0.5832	12.0257	0.0005

Odds Ratio Estimates			
Effect	Point Estimate	95% Wald Confidence Limits	
selfreport_fluidinta	7.556	2.409	23.695

Association of Predicted Probabilities and Observed Responses			
Percent Concordant	49.4	Somers' D	0.428
Percent Discordant	6.5	Gamma	0.766
Percent Tied	44.1	Tau-a	0.213
Pairs	1102	c	0.714

Self-reported urine frequency MODEL Afternoon vs. USG (Low cut-off)

The LOGISTIC Procedure

Model Information	
Data Set	WORK.FEMAALL
Response Variable	
Number of Response Levels	2
Model	binary logit
Optimization Technique	Fisher's scoring

Number of Observations Read	85
Number of Observations Used	76

Response Profile		
Ordered Value	USG_2nd_binary_low	Total Frequency
1	0	43
2	1	33

Probability modeled is USG_2nd_binary_low='0'.

Note: 9 observations were deleted due to missing values for the response or explanatory variables.

Model Convergence Status
Convergence criterion (GCONV=1E-8) satisfied.

Model Fit Statistics		
Criterion	Intercept Only	Intercept and Covariates
AIC	106.039	102.760
SC	108.369	107.421
-2 Log L	104.039	98.760

The LOGISTIC Procedure

Testing Global Null Hypothesis: BETA=0			
Test	Chi-Square	DF	Pr > ChiSq
Likelihood Ratio	5.2788	1	0.0216
Score	5.2195	1	0.0223
Wald	5.0859	1	0.0241

Analysis of Maximum Likelihood Estimates					
Parameter	DF	Estimate	Standard Error	Wald Chi-Square	Pr > ChiSq
Intercept	1	-0.2719	0.3318	0.6715	0.4125
missurinefreq	1	1.0827	0.4801	5.0859	0.0241

Odds Ratio Estimates			
Effect	Point Estimate	95% Wald Confidence Limits	
missurinefreq	2.953	1.152	7.566

Association of Predicted Probabilities and Observed Responses			
Percent Concordant	40.0	Somers' D	0.264
Percent Discordant	13.5	Gamma	0.494
Percent Tied	46.5	Tau-a	0.132
Pairs	1419	c	0.632

Self-reported first Afternoon urine volume MODEL Moring vs. USG (Low cut-off)

The LOGISTIC Procedure

Model Information	
Data Set	WORK.FEMAALL
Response Variable	
Number of Response Levels	2
Model	binary logit
Optimization Technique	Fisher's scoring

Number of Observations Read	85
Number of Observations Used	76

Response Profile		
Ordered Value	USG_2nd_binary_low	Total Frequency
1	0	43
2	1	33

Probability modeled is USG_2nd_binary_low='0'.

Note: 9 observations were deleted due to missing values for the response or explanatory variables.

Model Convergence Status
Convergence criterion (GCONV=1E-8) satisfied.

Model Fit Statistics		
Criterion	Intercept Only	Intercept and Covariates
AIC	106.039	98.784
SC	108.369	103.445
-2 Log L	104.039	94.784

The LOGISTIC Procedure

Testing Global Null Hypothesis: BETA=0			
Test	Chi-Square	DF	Pr > ChiSq
Likelihood Ratio	9.2551	1	0.0023
Score	9.0514	1	0.0026
Wald	8.6352	1	0.0033

Analysis of Maximum Likelihood Estimates					
Parameter	DF	Estimate	Standard Error	Wald Chi-Square	Pr > ChiSq
Intercept	1	-0.4274	0.3319	1.6588	0.1978
Black2_volume_250mL_	1	1.4571	0.4958	8.6352	0.0033

Odds Ratio Estimates			
Effect	Point Estimate	95% Wald Confidence Limits	
Black2_volume_250mL_	4.293	1.625	11.346

Association of Predicted Probabilities and Observed Responses			
Percent Concordant	45.4	Somers' D	0.348
Percent Discordant	10.6	Gamma	0.622
Percent Tied	44.0	Tau-a	0.173
Pairs	1419	c	0.674

Self-reported 3D urine color chary MODEL Afternoon vs. USG (Low cut-off)

The LOGISTIC Procedure

Model Information	
Data Set	WORK.FEMAALL
Response Variable	
Number of Response Levels	2
Model	binary logit
Optimization Technique	Fisher's scoring

Number of Observations Read	85
Number of Observations Used	76

Response Profile		
Ordered Value	USG_2nd_binary_low	Total Frequency
1	0	43
2	1	33

Probability modeled is USG_2nd_binary_low='0'.

Note: 9 observations were deleted due to missing values for the response or explanatory variables.

Model Convergence Status
Convergence criterion (GCONV=1E-8) satisfied.

Model Fit Statistics		
Criterion	Intercept Only	Intercept and Covariates
AIC	106.039	97.168
SC	108.369	101.829
-2 Log L	104.039	93.168

The LOGISTIC Procedure

Testing Global Null Hypothesis: BETA=0			
Test	Chi-Square	DF	Pr > ChiSq
Likelihood Ratio	10.8710	1	0.0010
Score	10.7289	1	0.0011
Wald	9.8781	1	0.0017

Analysis of Maximum Likelihood Estimates					
Parameter	DF	Estimate	Standard Error	Wald Chi-Square	Pr > ChiSq
Intercept	1	0.8109	0.3005	7.2843	0.0070
UC_3D_afternoon_bina	1	-1.6982	0.5403	9.8781	0.0017

Odds Ratio Estimates			
Effect	Point Estimate	95% Wald Confidence Limits	
UC_3D_afternoon_bina	0.183	0.063	0.528

Association of Predicted Probabilities and Observed Responses			
Percent Concordant	43.1	Somers' D	0.352
Percent Discordant	7.9	Gamma	0.691

4-ITEM MODEL Morning vs. USG (High cutoff)

The LOGISTIC Procedure

Model Information	
Data Set	WORK.FEMAALL
Response Variable	[illegible]
Number of Response Levels	2
Model	binary logit
Optimization Technique	Fisher's scoring

Number of Observations Read	85
Number of Observations Used	80

Response Profile		
Ordered Value	usgfirstbinaryhigh	Total Frequency
1	0	72
2	1	8

Probability modeled is usgfirstbinaryhigh=0.

Note: 5 observations were deleted due to missing values for the response or explanatory variables.

Model Convergence Status
Convergence criterion (GCONV=1E-8) satisfied.

Model Fit Statistics		
Criterion	Intercept Only	Intercept and Covariates
AIC	54.013	51.223
SC	56.395	63.133
-2 Log L	52.013	41.223

The LOGISTIC Procedure

Testing Global Null Hypothesis: BETA=0			
Test	Chi-Square	DF	Pr > ChiSq
Likelihood Ratio	10.7900	4	0.0290
Score	9.9901	4	0.0406
Wald	7.9988	4	0.0916

Analysis of Maximum Likelihood Estimates					
Parameter	DF	Estimate	Standard Error	Wald Chi-Square	Pr > ChiSq
Intercept	1	0.3082	1.4796	0.0434	0.8350
selfreport_fluidinta	1	0.00702	0.00658	1.1408	0.2855
selfreport_Urine_fre	1	1.7132	0.9700	3.1196	0.0774
Black1_volume_250mL_	1	1.3258	0.9842	1.8146	0.1780
Color_chart_3D_morni	1	-1.1179	0.9545	1.3717	0.2415

Odds Ratio Estimates			
Effect	Point Estimate	95% Wald Confidence Limits	
selfreport_fluidinta	1.007	0.994	1.020
selfreport_Urine_fre	5.547	0.829	37.128
Black1_volume_250mL_	3.765	0.547	25.914
Color_chart_3D_morni	0.327	0.050	2.123

Association of Predicted Probabilities and Observed Responses			
Percent Concordant	81.1	Somers' D	0.627
Percent Discordant	18.4	Gamma	0.630
Percent Tied	0.5	Tau-a	0.114
Pairs	576	c	0.813

Self-reported fluid intake MODEL Morning vs. USG (High cutoff)

The LOGISTIC Procedure

Model Information	
Data Set	WORK.FEMAALL
Response Variable	
Number of Response Levels	2
Model	binary logit
Optimization Technique	Fisher's scoring

Number of Observations Read	85
Number of Observations Used	80

Response Profile		
Ordered Value	usgfirstbinaryhigh	Total Frequency
1	0	72
2	1	8

Probability modeled is usgfirstbinaryhigh=0.

Note: 5 observations were deleted due to missing values for the response or explanatory variables.

Model Convergence Status
Convergence criterion (GCONV=1E-8) satisfied.

Model Fit Statistics		
Criterion	Intercept Only	Intercept and Covariates
AIC	54.013	52.776
SC	56.395	57.540
-2 Log L	52.013	48.776

The LOGISTIC Procedure

Testing Global Null Hypothesis: BETA=0			
Test	Chi-Square	DF	Pr > ChiSq
Likelihood Ratio	3.2375	1	0.0720
Score	2.7102	1	0.0997
Wald	2.7178	1	0.0992

Analysis of Maximum Likelihood Estimates					
Parameter	DF	Estimate	Standard Error	Wald Chi-Square	Pr > ChiSq
Intercept	1	1.0886	0.6800	2.5631	0.1094
selfreport_fluidinta	1	0.00923	0.00560	2.7178	0.0992

Odds Ratio Estimates			
Effect	Point Estimate	95% Wald Confidence Limits	
selfreport_fluidinta	1.009	0.998	1.020

Association of Predicted Probabilities and Observed Responses			
Percent Concordant	65.5	Somers' D	0.328
Percent Discordant	32.6	Gamma	0.335
Percent Tied	1.9	Tau-a	0.060
Pairs	576	c	0.664

Self-reported urine frequency MODEL Morning vs. USG (High cutoff)

The LOGISTIC Procedure

Model Information	
Data Set	WORK.FEMAALL
Response Variable	
Number of Response Levels	2
Model	binary logit
Optimization Technique	Fisher's scoring

Number of Observations Read	85
Number of Observations Used	81

Response Profile		
Ordered Value	usgfirstbinaryhigh	Total Frequency
1	0	72
2	1	9

Probability modeled is usgfirstbinaryhigh=0.

Note: 4 observations were deleted due to missing values for the response or explanatory variables.

Model Convergence Status
Convergence criterion (GCONV=1E-8) satisfied.

Model Fit Statistics		
Criterion	Intercept Only	Intercept and Covariates
AIC	58.511	55.482
SC	60.905	60.271
-2 Log L	56.511	51.482

The LOGISTIC Procedure

Testing Global Null Hypothesis: BETA=0			
Test	Chi-Square	DF	Pr > ChiSq
Likelihood Ratio	5.0289	1	0.0249
Score	4.9304	1	0.0264
Wald	4.1440	1	0.0418

Analysis of Maximum Likelihood Estimates					
Parameter	DF	Estimate	Standard Error	Wald Chi-Square	Pr > ChiSq
Intercept	1	1.3863	0.4226	10.7621	0.0010
selfreport_Urine_fre	1	1.7047	0.8374	4.1440	0.0418

Odds Ratio Estimates			
Effect	Point Estimate	95% Wald Confidence Limits	
selfreport_Urine_fre	5.500	1.065	28.392

Association of Predicted Probabilities and Observed Responses			
Percent Concordant	47.5	Somers' D	0.389
Percent Discordant	8.6	Gamma	0.692
Percent Tied	43.8	Tau-a	0.078
Pairs	648	c	0.694

Self-reported first morning urine volume MODEL Moring vs. USG (High cutoff)

The LOGISTIC Procedure

Model Information	
Data Set	WORK.FEMAALL
Response Variable	
Number of Response Levels	2
Model	binary logit
Optimization Technique	Fisher's scoring

Number of Observations Read	85
Number of Observations Used	81

Response Profile		
Ordered Value	usgfirstbinaryhigh	Total Frequency
1	0	72
2	1	9

Probability modeled is usgfirstbinaryhigh=0.

Note: 4 observations were deleted due to missing values for the response or explanatory variables.

Model Convergence Status
Convergence criterion (GCONV=1E-8) satisfied.

Model Fit Statistics		
Criterion	Intercept Only	Intercept and Covariates
AIC	58.511	58.949
SC	60.905	63.738
-2 Log L	56.511	54.949

The LOGISTIC Procedure

Testing Global Null Hypothesis: BETA=0			
Test	Chi-Square	DF	Pr > ChiSq
Likelihood Ratio	1.5615	1	0.2114
Score	1.8242	1	0.1768
Wald	1.7129	1	0.1906

Analysis of Maximum Likelihood Estimates					
Parameter	DF	Estimate	Standard Error	Wald Chi-Square	Pr > ChiSq
Intercept	1	1.2992	0.6513	3.9789	0.0461
Black1_volume_250mL_	1	1.0199	0.7793	1.7129	0.1906

Odds Ratio Estimates			
Effect	Point Estimate	95% Wald Confidence Limits	
Black1_volume_250mL_	2.773	0.602	12.772

Association of Predicted Probabilities and Observed Responses			
Percent Concordant	28.2	Somers' D	0.181
Percent Discordant	10.2	Gamma	0.470
Percent Tied	61.6	Tau-a	0.036
Pairs	648	c	0.590

Self-reported 3D urine color chary MODEL Morning vs. USG (High cutoff)

The LOGISTIC Procedure

Model Information	
Data Set	WORK.FEMAALL
Response Variable	
Number of Response Levels	2
Model	binary logit
Optimization Technique	Fisher's scoring

Number of Observations Read	85
Number of Observations Used	81

Response Profile		
Ordered Value	usgfirstbinaryhigh	Total Frequency
1	0	72
2	1	9

Probability modeled is usgfirstbinaryhigh=0.

Note: 4 observations were deleted due to missing values for the response or explanatory variables.

Model Convergence Status
Convergence criterion (GCONV=1E-8) satisfied.

Model Fit Statistics		
Criterion	Intercept Only	Intercept and Covariates
AIC	58.511	56.165
SC	60.905	60.954
-2 Log L	56.511	52.165

The LOGISTIC Procedure

Testing Global Null Hypothesis: BETA=0			
Test	Chi-Square	DF	Pr > ChiSq
Likelihood Ratio	4.3456	1	0.0371
Score	4.2042	1	0.0403
Wald	3.6081	1	0.0575

Analysis of Maximum Likelihood Estimates					
Parameter	DF	Estimate	Standard Error	Wald Chi-Square	Pr > ChiSq
Intercept	1	3.0445	0.7237	17.6956	<.0001
Color_chart_3D_morni	1	-1.5892	0.8367	3.6081	0.0575

Odds Ratio Estimates			
Effect	Point Estimate	95% Wald Confidence Limits	
Color_chart_3D_morni	0.204	0.040	1.052

Association of Predicted Probabilities and Observed Responses			
Percent Concordant	45.4	Somers' D	0.361
Percent Discordant	9.3	Gamma	0.661
Percent Tied	45.4	Tau-a	0.072
Pairs	648	c	0.681

4-ITEM MODEL Afternoon vs. USG (High cutoff)

The LOGISTIC Procedure

Model Information	
Data Set	WORK.FEMAALL
Response Variable	
Number of Response Levels	2
Model	binary logit
Optimization Technique	Fisher's scoring

Number of Observations Read	85
Number of Observations Used	67

Response Profile		
Ordered Value	usgsecondbinaryhigh	Total Frequency
1	0	57
2	1	10

Probability modeled is usgsecondbinaryhigh=0.

Note: 18 observations were deleted due to missing values for the response or explanatory variables.

Model Convergence Status
Convergence criterion (GCONV=1E-8) satisfied.

Model Fit Statistics		
Criterion	Intercept Only	Intercept and Covariates
AIC	58.469	50.498
SC	60.674	61.521
-2 Log L	56.469	40.498

The LOGISTIC Procedure

Testing Global Null Hypothesis: BETA=0			
Test	Chi-Square	DF	Pr > ChiSq
Likelihood Ratio	15.9713	4	0.0031
Score	14.9041	4	0.0049
Wald	10.3254	4	0.0353

Analysis of Maximum Likelihood Estimates					
Parameter	DF	Estimate	Standard Error	Wald Chi-Square	Pr > ChiSq
Intercept	1	0.6239	0.8958	0.4851	0.4861
selfreport_fluidinta	1	0.9681	0.8054	1.4447	0.2294
missurinefreq	1	1.7565	0.9140	3.6931	0.0546
Black2_volume_250mL_	1	1.4524	0.9547	2.3145	0.1282
UC_3D_afternoon_bina	1	-1.3379	0.8536	2.4567	0.1170

Odds Ratio Estimates			
Effect	Point Estimate	95% Wald Confidence Limits	
selfreport_fluidinta	2.633	0.543	12.766
missurinefreq	5.792	0.966	34.738
Black2_volume_250mL_	4.273	0.658	27.758
UC_3D_afternoon_bina	0.262	0.049	1.398

Association of Predicted Probabilities and Observed Responses			
Percent Concordant	83.7	Somers' D	0.711
Percent Discordant	12.6	Gamma	0.738
Percent Tied	3.7	Tau-a	0.183
Pairs	570	c	0.855

Self-reported fluid intake MODEL Afternoon vs. USG (High cutoff)

The LOGISTIC Procedure

Model Information	
Data Set	WORK.FEMAALL
Response Variable	
Number of Response Levels	2
Model	binary logit
Optimization Technique	Fisher's scoring

Number of Observations Read	85
Number of Observations Used	67

Response Profile		
Ordered Value	usgsecondbinaryhigh	Total Frequency
1	0	57
2	1	10

Probability modeled is usgsecondbinaryhigh=0.

Note: 18 observations were deleted due to missing values for the response or explanatory variables.

Model Convergence Status
Convergence criterion (GCONV=1E-8) satisfied.

Model Fit Statistics		
Criterion	Intercept Only	Intercept and Covariates
AIC	58.469	57.210
SC	60.674	61.620
-2 Log L	56.469	53.210

The LOGISTIC Procedure

Testing Global Null Hypothesis: BETA=0			
Test	Chi-Square	DF	Pr > ChiSq
Likelihood Ratio	3.2589	1	0.0710
Score	3.4362	1	0.0638
Wald	3.1778	1	0.0746

Analysis of Maximum Likelihood Estimates					
Parameter	DF	Estimate	Standard Error	Wald Chi-Square	Pr > ChiSq
Intercept	1	1.0414	0.4749	4.8100	0.0283
selfreport_fluidinta	1	1.2611	0.7075	3.1778	0.0746

Odds Ratio Estimates			
Effect	Point Estimate	95% Wald Confidence Limits	
selfreport_fluidinta	3.529	0.882	14.122

Association of Predicted Probabilities and Observed Responses			
Percent Concordant	42.1	Somers' D	0.302
Percent Discordant	11.9	Gamma	0.558
Percent Tied	46.0	Tau-a	0.078
Pairs	570	c	0.651

Self-reported urine frequency MODEL Afternoon vs. USG (High cutoff)

The LOGISTIC Procedure

Model Information	
Data Set	WORK.FEMAALL
Response Variable	
Number of Response Levels	2
Model	binary logit
Optimization Technique	Fisher's scoring

Number of Observations Read	85
Number of Observations Used	76

Response Profile		
Ordered Value	usgsecondbinaryhigh	Total Frequency
1	0	66
2	1	10

Probability modeled is usgsecondbinaryhigh=0.

Note: 9 observations were deleted due to missing values for the response or explanatory variables.

Model Convergence Status
Convergence criterion (GCONV=1E-8) satisfied.

Model Fit Statistics		
Criterion	Intercept Only	Intercept and Covariates
AIC	61.185	58.411
SC	63.516	63.072
-2 Log L	59.185	54.411

The LOGISTIC Procedure

Testing Global Null Hypothesis: BETA=0			
Test	Chi-Square	DF	Pr > ChiSq
Likelihood Ratio	4.7743	1	0.0289
Score	4.5202	1	0.0335
Wald	3.8698	1	0.0492

Analysis of Maximum Likelihood Estimates					
Parameter	DF	Estimate	Standard Error	Wald Chi-Square	Pr > ChiSq
Intercept	1	1.2879	0.3994	10.3997	0.0013
missurinefreq	1	1.6299	0.8286	3.8698	0.0492

Odds Ratio Estimates			
Effect	Point Estimate	95% Wald Confidence Limits	
missurinefreq	5.103	1.006	25.890

Association of Predicted Probabilities and Observed Responses			
Percent Concordant	44.8	Somers' D	0.361
Percent Discordant	8.8	Gamma	0.672
Percent Tied	46.4	Tau-a	0.084
Pairs	660	c	0.680

Self-reported first Afternoon urine volume MODEL Moring vs. USG (High cutoff)

The LOGISTIC Procedure

Model Information	
Data Set	WORK.FEMAALL
Response Variable	
Number of Response Levels	2
Model	binary logit
Optimization Technique	Fisher's scoring

Number of Observations Read	85
Number of Observations Used	76

Response Profile		
Ordered Value	usgsecondbinaryhigh	Total Frequency
1	0	66
2	1	10

Probability modeled is usgsecondbinaryhigh=0.

Note: 9 observations were deleted due to missing values for the response or explanatory variables.

Model Convergence Status
Convergence criterion (GCONV=1E-8) satisfied.

Model Fit Statistics		
Criterion	Intercept Only	Intercept and Covariates
AIC	61.185	58.784
SC	63.516	63.446
-2 Log L	59.185	54.784

The LOGISTIC Procedure

Testing Global Null Hypothesis: BETA=0			
Test	Chi-Square	DF	Pr > ChiSq
Likelihood Ratio	4.4011	1	0.0359
Score	4.1455	1	0.0417
Wald	3.5862	1	0.0583

Analysis of Maximum Likelihood Estimates					
Parameter	DF	Estimate	Standard Error	Wald Chi-Square	Pr > ChiSq
Intercept	1	1.3218	0.3979	11.0339	0.0009
Black2_volume_250mL_	1	1.5686	0.8283	3.5862	0.0583

Odds Ratio Estimates			
Effect	Point Estimate	95% Wald Confidence Limits	
Black2_volume_250mL_	4.800	0.947	24.339

Association of Predicted Probabilities and Observed Responses			
Percent Concordant	43.6	Somers' D	0.345
Percent Discordant	9.1	Gamma	0.655
Percent Tied	47.3	Tau-a	0.080
Pairs	660	c	0.673

Self-reported 3D urine color chary MODEL Afternoon vs. USG (High cutoff)

The LOGISTIC Procedure

Model Information	
Data Set	WORK.FEMAALL
Response Variable	
Number of Response Levels	2
Model	binary logit
Optimization Technique	Fisher's scoring

Number of Observations Read	85
Number of Observations Used	76

Response Profile		
Ordered Value	usgsecondbinaryhigh	Total Frequency
1	0	66
2	1	10

Probability modeled is usgsecondbinaryhigh=0.

Note: 9 observations were deleted due to missing values for the response or explanatory variables.

Model Convergence Status
Convergence criterion (GCONV=1E-8) satisfied.

Model Fit Statistics		
Criterion	Intercept Only	Intercept and Covariates
AIC	61.185	55.914
SC	63.516	60.575
-2 Log L	59.185	51.914

The LOGISTIC Procedure

Testing Global Null Hypothesis: BETA=0			
Test	Chi-Square	DF	Pr > ChiSq
Likelihood Ratio	7.2715	1	0.0070
Score	7.8672	1	0.0050
Wald	6.5400	1	0.0105

Analysis of Maximum Likelihood Estimates					
Parameter	DF	Estimate	Standard Error	Wald Chi-Square	Pr > ChiSq
Intercept	1	2.7932	0.5948	22.0557	<.0001
UC_3D_afternoon_bina	1	-1.9059	0.7453	6.5400	0.0105

Odds Ratio Estimates			
Effect	Point Estimate	95% Wald Confidence Limits	
UC_3D_afternoon_bina	0.149	0.035	0.641

Association of Predicted Probabilities and Observed Responses			
Percent Concordant	52.0	Somers' D	0.442
Percent Discordant	7.7	Gamma	0.741
Percent Tied	40.3	Tau-a	0.102
Pairs	660	c	0.721
